# Fusing physics and AI enhances accuracy and diagnostic capabilities in air quality forecasting

**DOI:** 10.1093/nsr/nwag570

**Published:** 2026-09-10

**Authors:** Huangjian Wu, Zifa Wang, Yuanhang Zhang, Wei Lin, Xinwei Li, Meng Gao, Hao Sun, Dawei Zhang, Wei Wang, Xiao Tang, Jiang Zhu, Yu Song, Chaoqun Ma, Pingqing Fu, Hang Su, Yafang Cheng, Gregory R Carmichael

**Affiliations:** State Key Laboratory of Atmospheric Environment and Extreme Meteorology, Institute of Atmospheric Physics, Chinese Academy of Sciences, Beijing 100029, China; Earth System Numerical Simulation Science Center, Institute of Atmospheric Physics, Chinese Academy of Sciences, Beijing 100029, China; State Key Laboratory of Atmospheric Environment and Extreme Meteorology, Institute of Atmospheric Physics, Chinese Academy of Sciences, Beijing 100029, China; Earth System Numerical Simulation Science Center, Institute of Atmospheric Physics, Chinese Academy of Sciences, Beijing 100029, China; College of Earth and Planetary Sciences, University of Chinese Academy of Sciences, Beijing 100049, China; College of Environmental Sciences and Engineering, Peking University, Beijing 100871, China; School of Mathematical Sciences and Center for Statistical Science, Peking University, Beijing 100871, China; State Key Laboratory of Atmospheric Environment and Extreme Meteorology, Institute of Atmospheric Physics, Chinese Academy of Sciences, Beijing 100029, China; Department of Geography, Hong Kong Baptist University, Hong Kong 999077, China; Gaoling School of Artificial Intelligence, Renmin University of China, Beijing 100872, China; China National Environmental Monitoring Center, Beijing 100012, China; China National Environmental Monitoring Center, Beijing 100012, China; State Key Laboratory of Atmospheric Environment and Extreme Meteorology, Institute of Atmospheric Physics, Chinese Academy of Sciences, Beijing 100029, China; Earth System Numerical Simulation Science Center, Institute of Atmospheric Physics, Chinese Academy of Sciences, Beijing 100029, China; State Key Laboratory of Atmospheric Environment and Extreme Meteorology, Institute of Atmospheric Physics, Chinese Academy of Sciences, Beijing 100029, China; College of Earth and Planetary Sciences, University of Chinese Academy of Sciences, Beijing 100049, China; College of Environmental Sciences and Engineering, Peking University, Beijing 100871, China; Aerosol Chemistry Department, Max Planck Institute for Chemistry, Mainz 55128, Germany; Institute of Surface-Earth System Science, Tianjin University, Tianjin 300072, China; State Key Laboratory of Atmospheric Environment and Extreme Meteorology, Institute of Atmospheric Physics, Chinese Academy of Sciences, Beijing 100029, China; Aerosol Chemistry Department, Max Planck Institute for Chemistry, Mainz 55128, Germany; Center for Global and Regional Environmental Research, University of Iowa, Iowa City, IA 52242, USA

**Keywords:** physics–AI coupling, air quality forecasting, model interpretability, PM_2.5_ and O_3_ prediction, uncertainty quantification, physical consistency

## Abstract

Accurate air quality forecasts with diagnostic value are pivotal for emission-control policies and public health, yet chemical transport models (CTMs) are hampered by uncertainties in physicochemical parameterizations, whereas AI-only approaches sacrifice interpretability for predictive power. Here, we introduce ProNet, a coupled forecasting framework that embeds observation-constrained neural correctors directly into the governing equations of CTMs. Rather than treating AI as an external postprocessor or a process-replacing surrogate, ProNet integrates trainable networks into physical simulations to systematically reduce process-level uncertainties, suppress error propagation and maintain physical constraints of mass transport. Applied to 2022–23 data across 338 Chinese cities, ProNet consistently outperforms physical CTMs and AI-only baselines, and better generalizes to extreme events beyond the training data. It also surpasses forecaster-adjusted operational forecasts, which often improve upon raw CTM guidance in severe episodes, improving accuracy by 6.1% for PM_2.5_ and 3.0% for O_3_. Notably, ProNet provides error bookkeeping, allowing forecasters to quantitatively attribute simulation errors to specific space and time. In-depth analysis of these process errors reveals systematic biases in current CTM parameterizations, providing actionable insights to refine the physical and chemical modules of numerical models. These results highlight the promise of physics–AI coupling for advancing air quality forecasting and deepening our understanding of the complex Earth system.

## Teaser

A novel physics–AI coupled framework, ProNet, embeds neural correctors directly into the governing equations of chemical transport models, outperforming both conventional models and human-adjusted operational forecasts in air quality forecasting while quantitatively diagnosing uncertainties in atmospheric chemical mechanisms.

## INTRODUCTION

Accurate and interpretable forecasting of airborne pollutants such as PM_2.5_ and O_3_ is essential for protecting public health and informing environmental policies. Reliable predictions help individuals reduce exposures [1,2], support timely warnings and emission-control actions by regulators [3] and inform the planning of intensive field campaigns for researchers [4]. Given the significant socioeconomic implications involved, it is crucial that forecasting systems deliver consistent performance across varied weather scenarios, reliably detect extreme pollution events and offer transparent interpretations, allowing experts to trace pollutant origins and assess predictive uncertainty.

Process-based chemical transport models (CTMs), commonly employed in operational forecasting systems worldwide [5], simulate transport, emissions, chemical reactions and deposition of air pollutants. Their explicit representation of atmospheric processes provides interpretability, facilitating source apportionment to pinpoint the timing, location and sectoral origins of pollutants [6] and enabling attribution of air quality improvements to factors such as

meteorological variability or emission reductions [7]. CTMs also support diagnostic analysis of forecast failures by tracking uncertainties in inputs and process parameterizations [8,9], thereby guiding model improvements [10]. Nonetheless, CTM accuracy remains hampered by imperfect emission inventories [11], which alone may account for >90% of simulation bias in some cases [12], and incomplete knowledge of physicochemical processes, leading to significant variations in predicted PM_2.5_ composition [13].

The rise of purely AI-based forecasting models has recently transformed numerical weather prediction (NWP) [14,15], with several data-driven systems now rivaling or surpassing traditional physics-based alternatives [14]. In air quality forecasting, data-driven models have also shown promising skills across various applications [16], such as attention-based PM_2.5_ spatiotemporal prediction [17] and transfer-learning-based O_3_ prediction [18]. However, their operational and mechanism-oriented application remains more challenging than in NWP because air pollution is heavily influenced by highly uncertain anthropogenic emissions, complex nonlinear multiphase chemistry, the sparsity of observations for rare high-pollution events and abrupt local control measures. Unlike weather systems driven largely by stable solar radiation, air pollution stems heavily from volatile and poorly quantified anthropogenic emissions [19,20]. The combined challenges of emission uncertainties, complex chemical interactions and limited observational data hinder the interpretability and generalizability of purely AI-based approaches, particularly for risk-sensitive applications or mechanism-focused studies [21]. Even advanced systems like Aurora [22], while showing encouraging skill for surface pollutants, remain constrained by limited training data, highly skewed pollutant-concentration distributions spanning clean background conditions to episodic high-PM_2.5_ and high-O_3_ events and intricate precursor-emission patterns.

Hybrid approaches that integrate physical modeling with AI have thus attracted growing interest [23,24]. Such integrations aim to enhance predictive skill while retaining interpretability and mechanistic insight [25]. Recent examples include deep learning enhanced with physical advection schemes (NowcastNet) [26] and differentiable solvers augmented with machine-learning modules (NeuralGCM) [27]. However, transitioning these emerging techniques to high-resolution atmospheric chemistry forecasting remains hindered by fundamental computational and mathematical bottlenecks. For instance, physics-informed machine learning (ML) relying on soft constraints [28] often struggles to converge when optimizing stiff chemical kinetics, while fully differentiable 3D CTMs [29] face prohibitive memory overheads for backpropagation. Consequently, in the domain of air quality forecasting, current frameworks mostly retreat to simpler integrations. AI-based postprocessing techniques do improve predictive skill [30]; however, they provide little clarity about where, when, or through which processes prediction errors arise. Alternatively, replacing certain physical submodules with AI-based emulators can reduce computational expense [31], yet when these surrogates are trained exclusively on synthetic model outputs rather than real-world observations, they inevitably inherit the limits of their parent CTMs.

To overcome the persistent trade-off between accuracy and interpretability, we introduce ProNet: a tightly coupled forecasting framework that embeds observation-constrained neural correctors directly into the governing equations of CTM. Rather than treating AI as an external postprocessor or module replacement, ProNet employs trainable networks to systematically mitigate process-level uncertainties within the physical simulation, thereby curtailing error propagation, preserving physical consistency and illuminating systemic model deficiencies. Evaluations across 338 Chinese cities confirm that ProNet exceeds existing physical and AI-only models in predicting PM_2.5_ and O_3_ generalizes robustly to extreme events, and surpasses forecaster-adjusted operational forecasts under the same lead-time and daily-mean evaluation protocol. This comparison provides a stringent human-in-the-loop benchmark because forecasters often improve upon raw CTM guidance during severe pollution episodes. More importantly, it identifies when and where conventional models fail, revealing critical gaps in current understandings of PM_2.5_ formation mechanisms. By uniting physical modeling with trainable correctors in a unified architecture, ProNet provides both state-of-the-art forecasts and deeper insights into air quality forecasting, paving the way for a new generation of intelligible, high-fidelity Earth system modeling.

## THE ProNet FRAMEWORK

The ProNet framework is designed based on the governing equation of air quality forecasting, which could deduce that the CTM forecasting error can be decomposed into the error transported from the previous time step, and the newly generated error stemming from the uncertainties in the processes of CTM (see Materials and Methods). As shown in Fig. 1a, ProNet corrects the conventional CTM with an error transport model (ETM). The baseline CTM employed in this study is the Nested Air Quality Prediction Modeling System (NAQPMS), a widely adopted system in operational air quality forecasting [32]. Technical specifications and model configurations are detailed in Section S2.1. The ETM dynamically corrects baseline predictions by numerically solving the governing equations for CTM errors. The ETM combines a process-based transport module that is methodologically aligned with the CTM (see Section S2.3) with two neural-network correctors.

**Figure 1.**
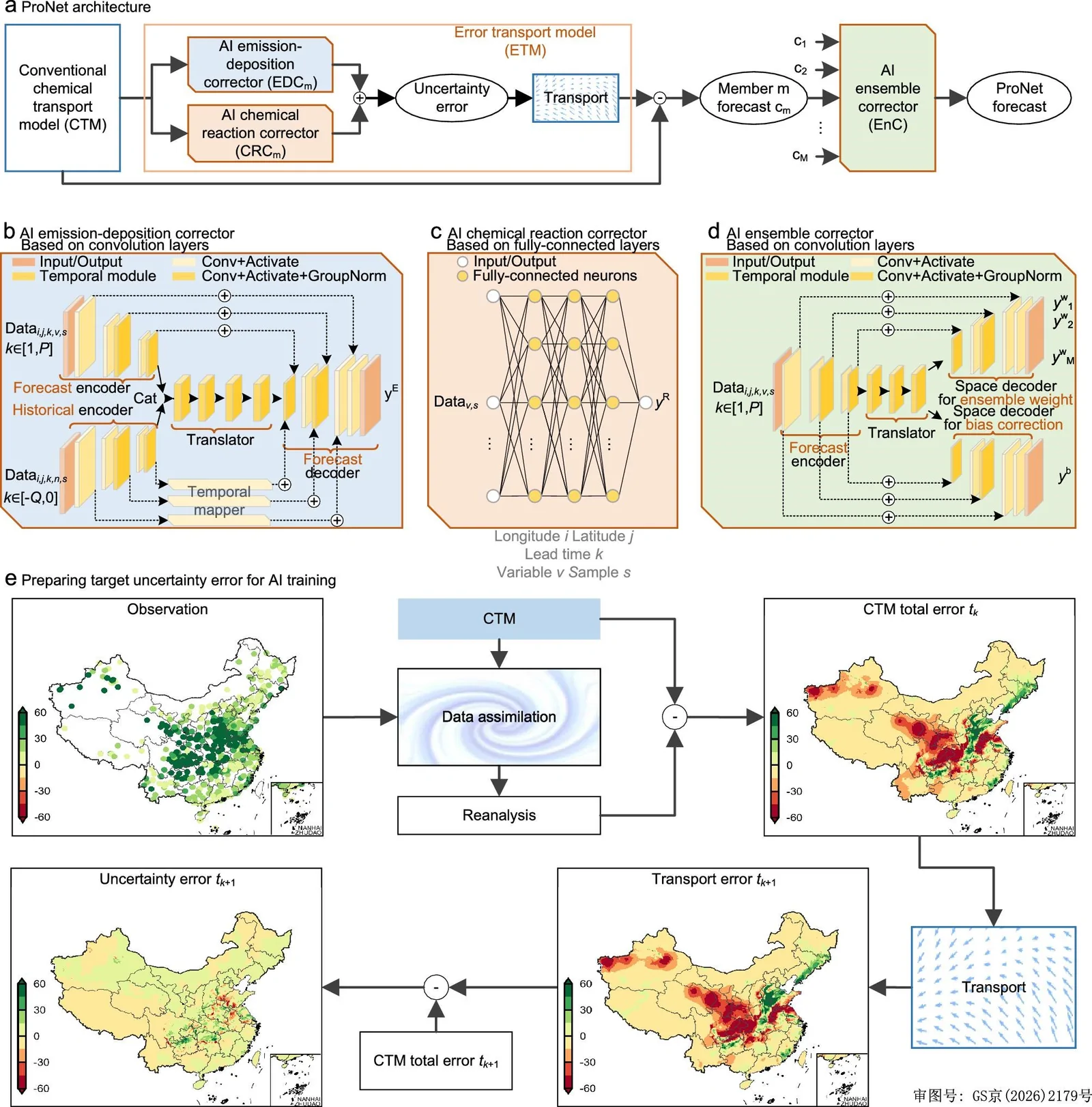
Architecture and training framework of ProNet. (a) Schematic of the ProNet forecasting framework. ProNet integrates a CTM for baseline predictions with an ETM to refine forecasts. The ETM combines a physical process module (transportation) with two neural networks: (i) the emission-deposition corrector, which learns spatiotemporal patterns to compensate for errors arising from emission and deposition processes in the CTM; and (ii) the chemical-reaction corrector, which captures interactions among chemical species to correct inaccuracies in the chemical reaction processes. The outputs from the CTM and multiple ETMs are merged to generate member forecasts, which are subsequently processed by the ensemble corrector to mitigate the accumulation of biases stemming from AI correctors in ETM. (b–d) Architectural schematics of the emission-deposition corrector (b), chemical-reaction corrector (c) and ensemble corrector (d). (e) Techniques to generate target uncertainty error for the training of AI correctors in ETM.

Specifically, ProNet features two neural correctors to address distinct error sources: the emission-deposition corrector, which utilizes historical errors and meteorological states to correct general spatiotemporal uncertainties, and the chemical-reaction corrector, which processes multi-species concentrations to correct PM_2.5_ errors from chemical parameterizations (details in Section S2.2). To train these correctors, the transport module isolates target uncertainty errors from overall CTM forecast errors by subtracting the computed transported error (Equation 2) from the total forecast error at each step (Fig. 1e).

The ensemble corrector detects and suppresses bias accumulation that would otherwise increase by orders of magnitude during forecasting. While structurally similar to the emission-deposition corrector, it employs one spatial encoder and two distinct spatial decoders, one for ensemble weights and another for bias estimation (Fig. 1d). The final ProNet forecast is computed as the weighted average of ensemble forecasts plus the estimated ensemble bias. Further details regarding data assimilation, network architectures, training techniques for the ProNet approach are elaborated in Sections S2 and S3.

Additionally, future meteorological forecast states (such as wind speed, wind direction and boundary layer height) are incorporated as input features to help the correctors adjust to varying meteorological regimes. The framework’s robustness against wind-field errors is primarily achieved by the 1-h short-step cyclic data assimilation at the training stage, which resets advection displacement biases at each hourly step, and the hourly error decomposition that isolates transported errors from local uncertainties. Nevertheless, this physics-informed approach maintains the physical constraints of process-driven modules while offering new insights into these atmospheric processes.

In this framework, the diagnostic interpretability is distinctly defined across three levels. First, process-constrained correction is achieved by maintaining the physical consistency of mass transport. Second, interpretable error bookkeeping provides spatiotemporal source apportionment of forecast errors, highlighting specific regions and periods for priority model improvement. Third, for mechanistic inference, while ProNet cannot provide explicit chemical reaction pathways, it dissects statistical associations between component-specific uncertainties, including nitrate, sulfate, ammonia, SOA and OC, and total PM_2.5_ forecast errors at a sub-grid scale.

## RESULTS

### Interpretable analysis of the coupled model

The ProNet framework introduces an interpretable architecture that simultaneously addresses two critical challenges in air quality modeling, namely diagnosing the sources of forecasting errors and elucidating deficiencies in PM_2.5_ formation mechanisms. Unlike conventional approaches that rely on costly sensitivity analyses or ambient PM_2.5_ component observations [33], our coupled model systematically quantifies both uncertainty errors and initial condition (IC) errors through a source apportionment technique [34] tagging error propagation, while also disentangling the complex physicochemical processes governing aerosol formation. This dual capability provides actionable insights for refining emission inventories and advancing mechanistic understanding.

Error apportionment analysis reveals the source of CTM errors across temporal and spatial scales, as illustrated by a pollution episode in Beijing’s January 2023 (Fig. 2a and c). While the CTM successfully captured the pollution episode’s temporal evolution with peak concentrations exceeding 100 μg m^−^^3^, it exhibited systematic overestimation (Fig. 2d). Temporal error apportionment reveals that the errors on 13 January mainly originated from accumulated uncertainty errors propagating from the prior 4 days (Fig. 2b). Spatial attribution analysis further attributes the 15 January PM_2.5_ overestimation in Beijing to the comprehensive model forecast errors (confounding local emission uncertainties, meteorological transport biases and boundary layer simulation errors) originating from the upwind Shanxi and Shandong provinces (Fig. 2c). By tracking regional source tags, we identified a transport trajectory where simulated upwind errors accumulated and propagated downwind (detailed in Section S9 and Video S1).

**Figure 2.**
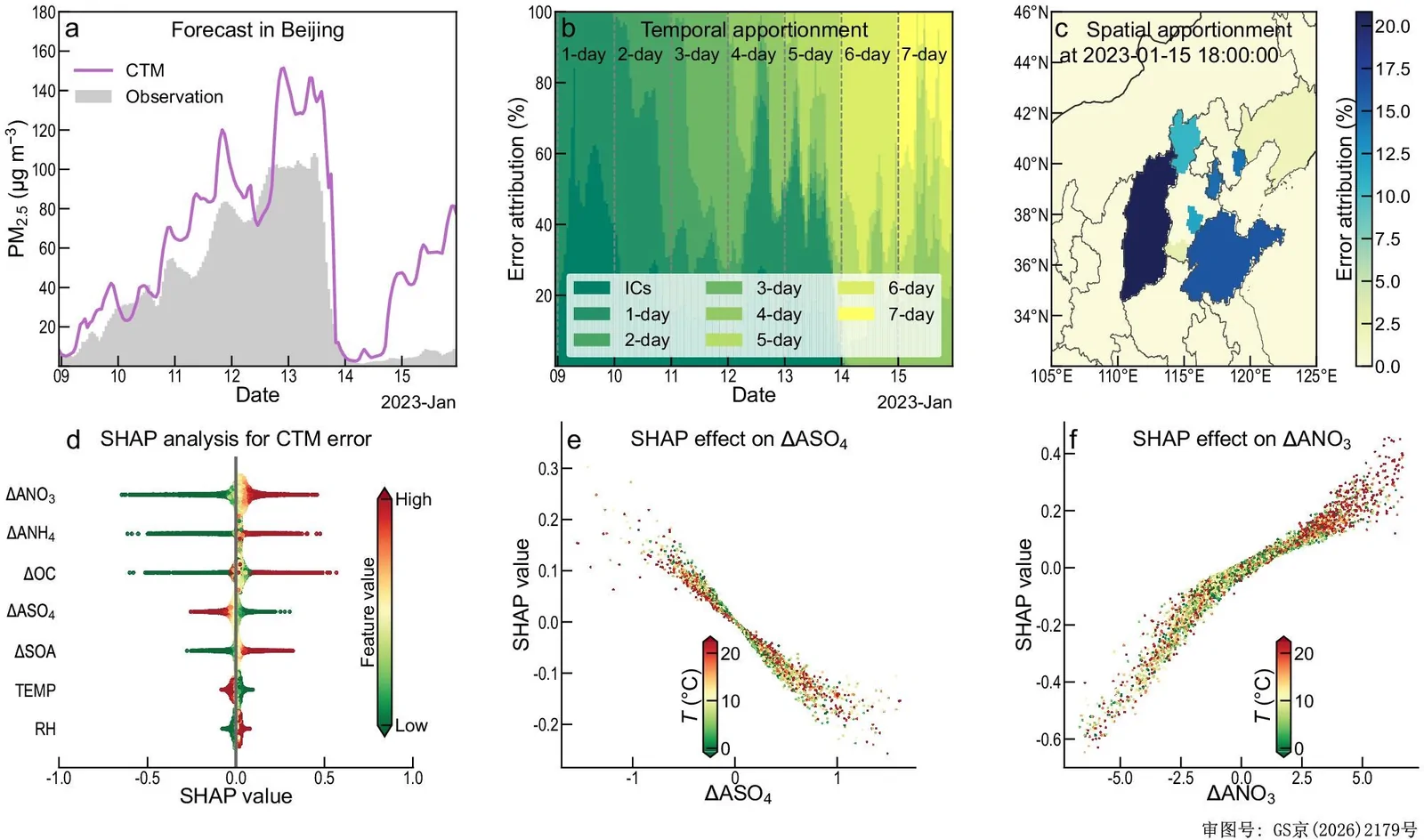
Interpretable analysis of PM_2.5_ forecasting. (a–c) Error apportionment for a forecast in Beijing. An animated version of the error apportionment is provided in Video S1. (a) is the forecast to analysis and panel (b) is the temporal error apportionment identifying when the total error is stemming from, including the ICs (before the forecast), and uncertainties of CTM in 1–7 day forecasts. Panel (c) is the spatial error apportionment identifying where the total forecast error in Beijing at 15 January 2023 18:00:00 is stemming from. (d–f) Uncertainty analysis of PM_2.5_ formation mechanisms. (d) Beeswarm plots of key drivers influencing PM_2.5_ concentration errors. Each point represents an individual sample at a specific grid and time step, where its *x* axis, i.e. the SHAP values represent the impact of predictors on CTM forecast error for PM_2.5_. Point density is visualized through vertical stacking. An increase in SHAP value alongside the concentration of a PM_2.5_ component indicates an overestimation in the formation of that component, while a decrease suggests an underestimation. (e, f) SHAP dependence plots reveal parameter-specific patterns for sulfate (e) and nitrate (f). The SHAP value increases with the rise in ΔANO_3_ and decreases with ΔASO_4_, indicating an overestimation of nitrate formation and an underestimation of sulfate formation. A pronounced temperature effect is evident in panel (e), revealing a greater underestimation of sulfate formation during winter.

Concurrently, the model investigates PM_2.5_ formation processes by isolating statistical correlations of component-specific uncertainties, including nitrate, sulfate, ammonia, secondary organic aerosols and organic carbon, using observations of total PM_2.5_ concentrations (technical details in Section S4). Experimental studies report uptake coefficients for NO_2_, nitrate radicals (NO_3_) and dinitrogen pentoxide (N_2_O_5_) spanning orders of magnitude under varying conditions [35–37]. As demonstrated by Fig. 2d and f, elevated nitrate levels correlate with increased CTM forecast errors, suggesting a potential systematic overestimation of nitrate formation. This aligns with observational evidence showing pronounced overestimation of nitrate levels in CTM simulations [38]. Crucially, this hypothesis is strongly supported by independent external validation from the MICS-Asia III multi-model comparison, which reveals that current CTMs systematically overestimate nitrate production (exhibiting artificially high nitric oxidation ratios) while underestimating sulfate concentrations across East Asia [13]. We hypothesize that this bias may arise from unrepresented mechanisms such as nonvolatile cations in fine-mode aerosols artificially elevating aerosol pH and enhancing nitrate partitioning [39], or the absence of nitryl chloride formation from dinitrogen pentoxide uptake in current CTMs [40]. Importantly, these specific chemical pathways are subjective hypotheses based on domain expertise and statistical correlations rather than direct causal discoveries of ProNet, as SHapley Additive exPlanations (SHAP) values indicate feature importance rather than definitive causation. Nevertheless, by providing spatiotemporally continuous correlations between precursors and simulation errors, ProNet serves as a practical diagnostic reference for screening new chemical hypotheses. Our analysis also reveals a systematic underestimation of sulfate formation, particularly at lower temperatures (Fig. 2e), alongside an overestimation of secondary organic aerosol production (Fig. 2d).

Analogous to conventional source attribution and chemical mechanism analysis based on CTM, ProNet’s error attribution and chemical diagnostics provide valuable quantitative insights into process-level uncertainties under the standard assumption of prescribed meteorological inputs. A comprehensive uncertainty analysis (detailed in Section S13, and Figs S14 and S15) evaluates the numerical stability of the diagnostic patterns identified by ProNet, providing a practical uncertainty reference for model refinement.

### Mitigating bias accumulation through ensemble correction

Forecasting systems operate through recursive state predictions to extend forecast horizons. However, single-step model optimizations fail to ensure commensurate gains in multi-day forecasting accuracy. Even with a perfect model, small errors in the initial conditions can lead to widely diverging outcomes due to the chaotic behavior, also known as the butterfly effect [41]. While the ETM correctors reduce per-step uncertainties, they inadvertently induce small biases. Without proper handling, biases introduced at each time step accumulate in the total concentration, leading to substantial errors in long-lead-time forecasts. As shown in Fig. 3, the forecasts without an ensemble corrector exhibit little bias at the beginning. However, the bias increases nearly linearly over the first few days, leading to an ensemble spread exceeding 30 μg m^−3^ for lead times beyond 3 days.

**Figure 3.**
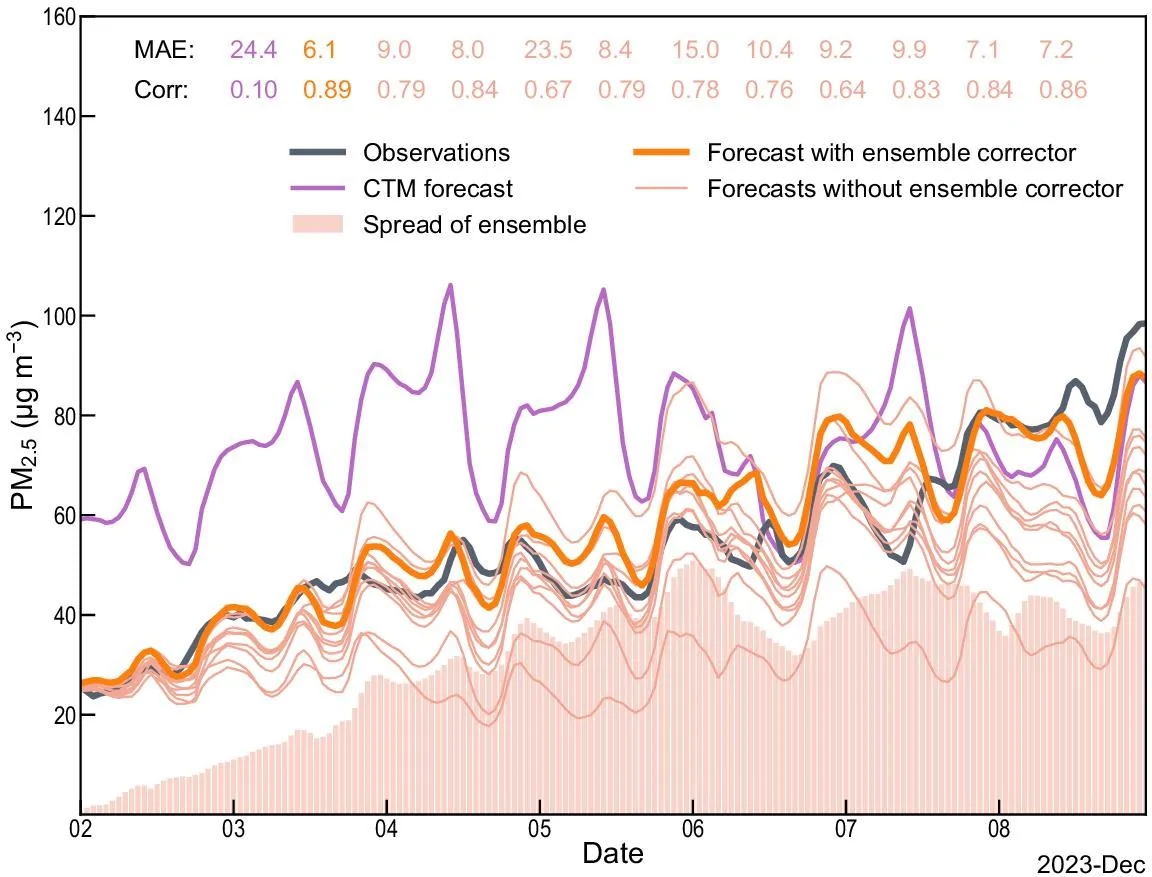
Suppressing bias accumulation via the ensemble corrector. The ChengYu Region in the Sichuan Basin, being relatively isolated from external pollution sources, functions as a semi-closed system ideal for assessing bias accumulation. Without ensemble correction, systematic biases from data-driven modules accumulate progressively, leading to an increase in ensemble spread by orders of magnitude. The proposed approach constructs a 10-member ensemble using neural network parameters sampled from different training epochs; these are subsequently processed through the ensemble corrector to detect and mitigate bias accumulation, thereby improving long-term forecast accuracy.

Deep learning-based forecasting models commonly face bias accumulation challenges stemming from two key sources. First, intrinsic model errors emerge from data noise and optimization constraints. Second, the iterative state estimation process in multi-step forecasting progressively amplifies these single step errors. While bias mitigation is feasible, the bias–variance trade-off [42] fundamentally constrains accuracy improvements. Furthermore, iterative effects during extended forecasting can magnify single-step biases by orders of magnitude, leading to substantial forecast degradation. The Pangu model counters this by employing multi-timescale frameworks to reduce computational cycles [14], effectively limiting bias accumulation. However, these systems require training parallel models for different time steps, which introduces two limitations: computational overhead and potential loss of high-frequency temporal patterns in coarse-grained models. To address this, we construct a 10-member ensemble of ETMs (using parameters from epochs 291–300) combined with an AI ensemble corrector. As shown in Fig. S21, while the pure AI model is less affected because it bypasses physical integration, the coupled model exhibits significant error accumulation across regions (BTH, YRD, PRD), with ensemble members diverging over time (Fig. S13). The ensemble corrector successfully identifies and mitigates this accumulation, bringing greater targeted improvement for the coupled model. Detailed procedures of model training and operational forecasting pipelines are provided in Section S7.

### Advantages of the coupled model over pure AI-based approaches

While AI-based models have demonstrated considerable potential in air quality forecasting, their ‘black-box’ nature often fails to ensure dynamical consistency in predictions. To overcome this limitation, the ProNet framework integrates AI-based modules with process-based components, guided by the governing equations of air quality forecasting. To evaluate its advantages, we compare ProNet against a purely AI-based version that removes all process-driven modules (see Fig. S1 and Section S2.2). Unlike Aurora [22], this purely AI-based counterpart ingests multi-time-step state inputs and learns spatiotemporal patterns both preceding and following the initial condition. Notably, it achieves lower root-mean-square error (RMSE) than four operational CTMs across 1- to 7-day forecast horizons (Fig. 5a).

The coupled model achieves significant error reduction across key economic regions, achieving an error reduction of 13.2% in the Beijing–Tianjin–Hebei and Surrounding Region (denoted as BTHS, including Beijing and surrounding cities), 5.4% in the Yangtze River Delta Region (denoted as YRD) and 8.9% in the Yangtze River Midstream City Cluster (denoted as YRM). The specific cities comprising these regions are comprehensively listed in Table S2.

Without physical constraints, purely data-driven models often produce unphysical results, such as non-physical concentration shifts and temporal noise. To quantitatively assess this, we introduced three relative diagnostics in Section S8 to evaluate dynamical resemblance against the process-based CTM baseline: relative mass-variation resemblance, temporal smoothness and transport realism (Fig. S4 and Video S3). We clarify that these metrics evaluate relative physical resemblance to the CTM’s dynamical core rather than absolute thermodynamic truth, as CTMs inherently incorporate discretization errors, sub-grid parameterizations and continuous hourly mass fluxes from emissions and deposition. The evaluation reveals that the pure AI model exhibits a relative mass imbalance index of 0.15 (max 0.59) and a temporal variance deviation of 0.07 (max 0.33) relative to the CTM. In contrast, ProNet maintains a significantly closer dynamical resemblance to the CTM’s physical core, reducing the relative mass imbalance to 0.11 (max 0.33) and temporal deviation to 0.03 (max 0.09). Moreover, ProNet achieves a transport realism correlation of 0.5 with the CTM-derived transport-only tendency fields, far exceeding the pure AI model (0.1). This stronger alignment with the CTM transport operator suggests physically coherent spatial transport, improving assimilation and forecasting under unseen scenarios.

Data assimilation techniques have been among the most important factors contributing to improved forecasting accuracy. With coupling, the ProNet approach assimilates observations into ICs and systematically reduces 1-day forecast RMSE across 97.3% of stations (Fig. 4a). As demonstrated in a sand and dust event case study (Fig. 4b and c and Video S2), the pure-AI-based model struggles to resolve short-term pollutant dynamics (≤24 h), while the coupled system exhibits enhanced observational influence. The coupled system successfully predicts the upcoming high PM_2.5_ concentrations in Beijing, achieving an RMSE 62% lower than that of the pure AI model.

**Figure 4.**
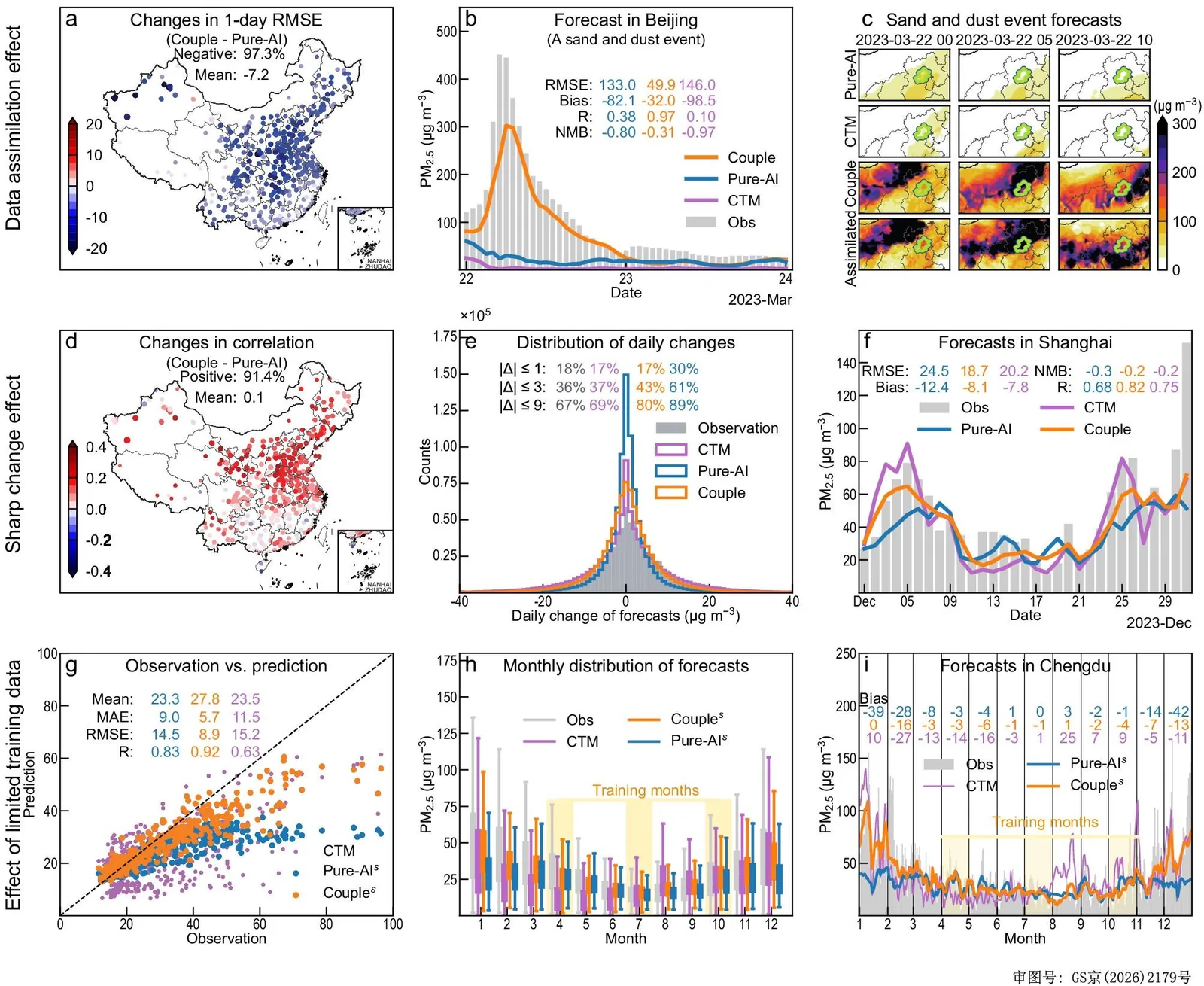
Performance advantages of the coupled model over pure AI-based approaches. (a–c) The coupled model improves short-term forecasts by effectively assimilates observations into ICs. (a) Distribution of 1-day RMSE changes by coupling. (b) A forecast case in Beijing. (c) PM_2.5_ distributions of forecasts during a sand and dust event. (d–f) The coupled model outperforms pure AI approaches in sharp pollution changes. (d) Distribution of correlation coefficient changes induced by coupling. (e) Distribution of daily PM_2.5_ concentration changes in the forecasts. (f) Forecasts for Guangzhou in January. (g–i) The coupled model exhibits better generalization with limited training data (April, July and October 2022). (g) Scatter plot of national daily mean PM_2.5_ concentrations in 2023. (h) Monthly distribution of forecasts. (i) Forecasts in Chengdu.

The pure AI model optimizes an aggregated loss function across all training samples, performing well for clean days under common conditions. However, policymaking prioritizes accurate predictions of polluted episodes and abrupt pollution changes, where our physics–AI coupled model demonstrates superior performance. It more accurately predicts daily concentration changes and achieves higher correlation coefficients across 91.4% of monitoring stations (Fig. 4d and f).

To test generalizability, we trained both models on data from 3 months (deliberately excluding winter). While performance was comparable, the pure AI model predicted national averaged PM_2.5_ levels below 40 μg m^−3^ for all days, whereas the coupled model captured higher concentrations in months outside the training period (Fig. 4g and i). The superior generalizability of the coupled model is critical for air quality forecasting, because the compounding effects of dynamic policy adjustments (e.g. the Air Pollution Prevention and Control Action Plan [43]) and climate variability [44] render historical training data insufficient to cover future scenarios. More discussions about sensitivity experiments on training data scarcity are provided in Section S11.

Furthermore, beyond data scarcity, we evaluated out-of-distribution generalization by applying models trained on 2022 data to predict 2023 conditions. The noticeable differences in pollution levels and distribution patterns between these two years provided a basis to assess model performance across various pollution levels, seasons, regions and special events such as dust storms and festival emissions. ProNet showed better adaptability to unseen high-pollution periods compared to the pure AI model, although both models still faced challenges with sudden human-driven emission spikes like fireworks. Detailed stratification analyses are provided in Section S14.

To justify the hybrid architecture, we evaluated an all-AI Corrector approach that corrects CTM outputs without a physical transport module. While incorporating CTM baseline simulation data achieves a significant performance improvement over pure AI models, its overall forecast performance remains inferior to ProNet (Fig. S21). Furthermore, the all-AI Corrector also underperforms compared to ProNet in terms of assimilation data utilization, concentration change prediction and performance under data-missing scenarios (Fig. S17, detailed in Section S10).

### Improved air quality forecasting

In ProNet, deep learning modules that correct uncertainties in CTMs were trained on 2022 forecasts and validated with 2023 data. These modules effectively capture spatial and temporal variations in uncertainty error (Fig. S3), reducing forecasting RMSE at 99.7% of cities and 99.2% days. While deep learning modules reduce uncertainty error by 8.2%, the coupled model achieves a 32.5% reduction in total error, indicating that the integration framework synergistically amplifies the improvements made by the deep learning modules.

An ablation study systematically evaluates the performance contributions of ProNet’s key components (Fig. S21). Integrating data assimilation and physical transport reduces the 1–7-day forecast RMSE by 8.6% compared to raw CTM. Adding the neural correctors in the ETM yields an additional 13.1% error reduction. Finally, applying the ensemble corrector suppresses bias accumulation, contributing another 15.2% error reduction, with benefits becoming more pronounced at longer lead times.

To benchmark against SOTA global AI models, we evaluated Aurora over China for 2023 (Fig. S5 and Section S12). Note that because Aurora does not output continuous hourly forecasts suitable for daily averages, this evaluation is based on hourly RMSE. Aurora’s overall PM2.5 forecast error is comparable to the CTM, while our pure AI baseline outperforms both. ProNet further reduces the hourly forecast error to 25.6 μg m^−^^3^, outperforming Aurora (32.6 μg m^−^^3^) by 21%. While Aurora’s use of global satellite data improves dust forecasting in northern regions, ProNet’s physics-coupled design and high-resolution station data yield superior accuracy across the vast majority of cities and lead times.

In China, human forecasters produce the official air quality forecasts by integrating CTM predictions with expert interpretation (see Section S6). This forecaster-adjusted product provides a stringent benchmark because it already improves upon raw CTM guidance during severe pollution episodes. To ensure a fair comparison, all methods are evaluated under the same D+1 to D+7 lead-time and daily-mean protocol, with hourly ProNet and CTM outputs aggregated to the same city-level daily means as the operational product.

As demonstrated in Fig. 5a, operational forecasts made by professional human forecasters across 338 cities achieve a PM_2.5_ RMSE of 21.2 μg m^−3^ (14.1%–18.9% lower than individual CTM). However, this expertise-driven approach presents inherent limitations: Subjective interpretation challenges quantification, while manual processing struggles with high-dimensional datasets and intricate spatiotemporal relationships. With data-driven modules learning from historical datasets, ProNet outperforms human forecasts with an 18.4% reduction in RMSE, achieving a 7-day RMSE lower than the 1-day RMSE by human forecasters. Accuracy rises nationally from 81.9% to 88.0% (Fig. 5c), with spatially heterogeneous gains (Fig. S7): 13.2% in BTHS, 5.4% in Yangtze River Delta Region (YRD), and 8.9% in Yangtze River Midstream City Cluster (YRM). The specific cities of the regions are listed in Table S2. More details about forecast accuracies in more regions are provided in Table S3.

**Figure 5.**
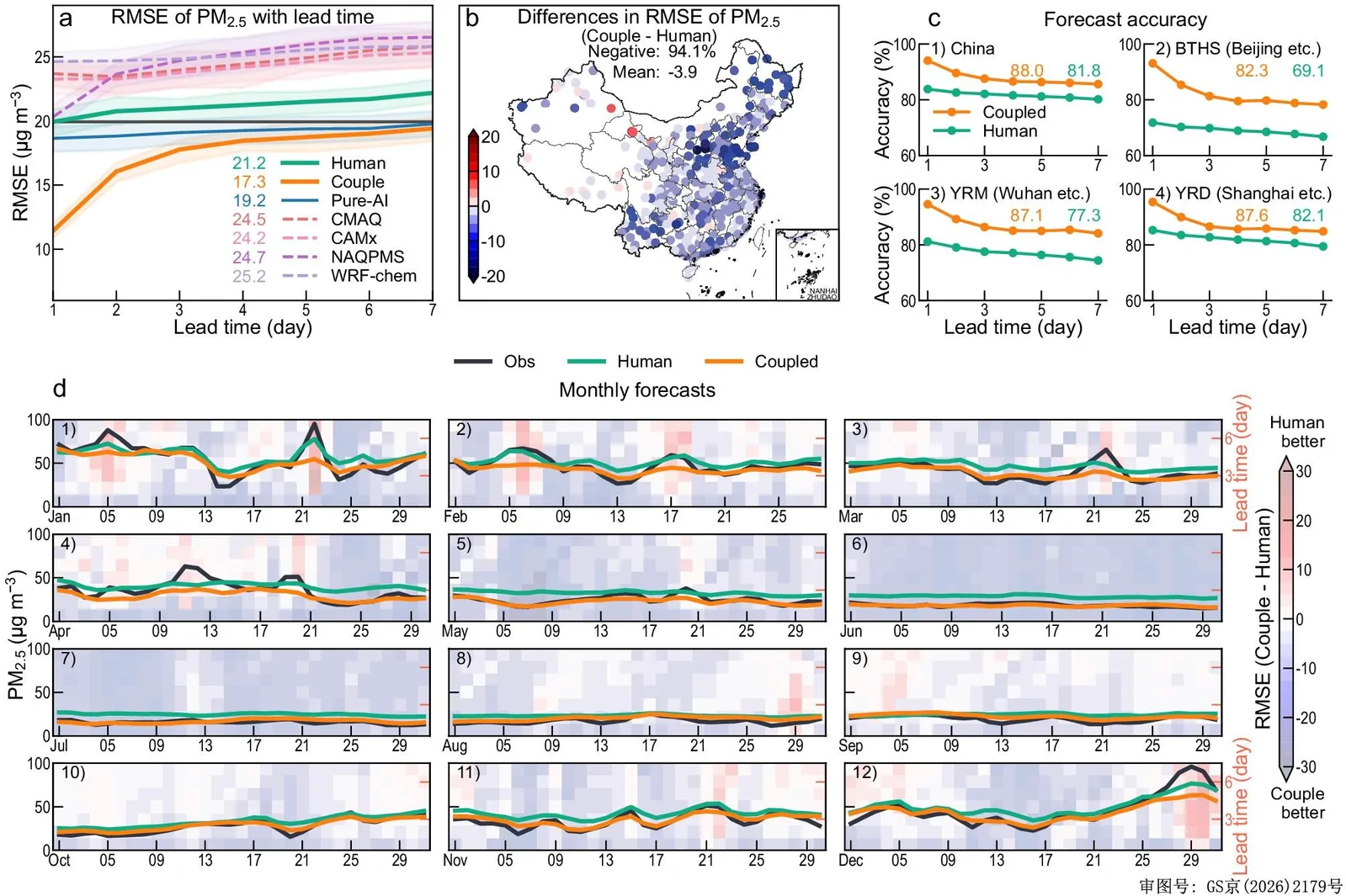
Performance comparison of the coupled model against operational forecasts over China. (a) RMSE curves as functions of lead time for city-level daily-mean PM_2.5_ across all monitored cities, with shaded regions denoting 95% confidence intervals. Human forecasts and model outputs are evaluated using the same daily-mean metric and lead-time definition. Numbers beside the legend indicate mean RMSE averaged across D+1 to D+7. (b) Spatial distributions of RMSE reduction achieved by the coupled model for PM_2.5_. (c) Forecasting accuracy of PM_2.5_ for China as a whole (1) and three regions (2–4). The specific cities of the regions are listed in Table S2, and the procedures for accuracy calculation is detailed in Section S5. (d) Monthly evaluation of PM_2.5_ forecasts during 2023: solid lines show observed and forecasted concentrations (left *y* axis), while background colors quantify RMSE differences between human and couple forecasts (right *y* axis). Collectively, these results demonstrate ProNet’s superior performance over both human forecasts and conventional CTMs in spatiotemporal coverage and prediction fidelity.

For pollution regulation, human forecasters strategically prioritize high-concentration events. As shown in Fig. 6b, the human predictions systematically produce higher concentrations, yielding a national average 16.1% higher than observations, which helps them achieve a higher hit rate for severe pollution levels. To address this, we developed a fine-tuned variant, Couple^H^, sharing the same ETM correctors but utilizing an ensemble corrector trained with a customized loss function that upweights higher pollution levels (Section S2.2). Compared to the standard model, this variant slightly reduces overall accuracy by 0.2% but improves polluted-day accuracy (PM_2.5_ > 75 μg m^−3^) by 12.6% (Fig. 6), demonstrating its high practical value for pollution warning.

**Figure 6.**
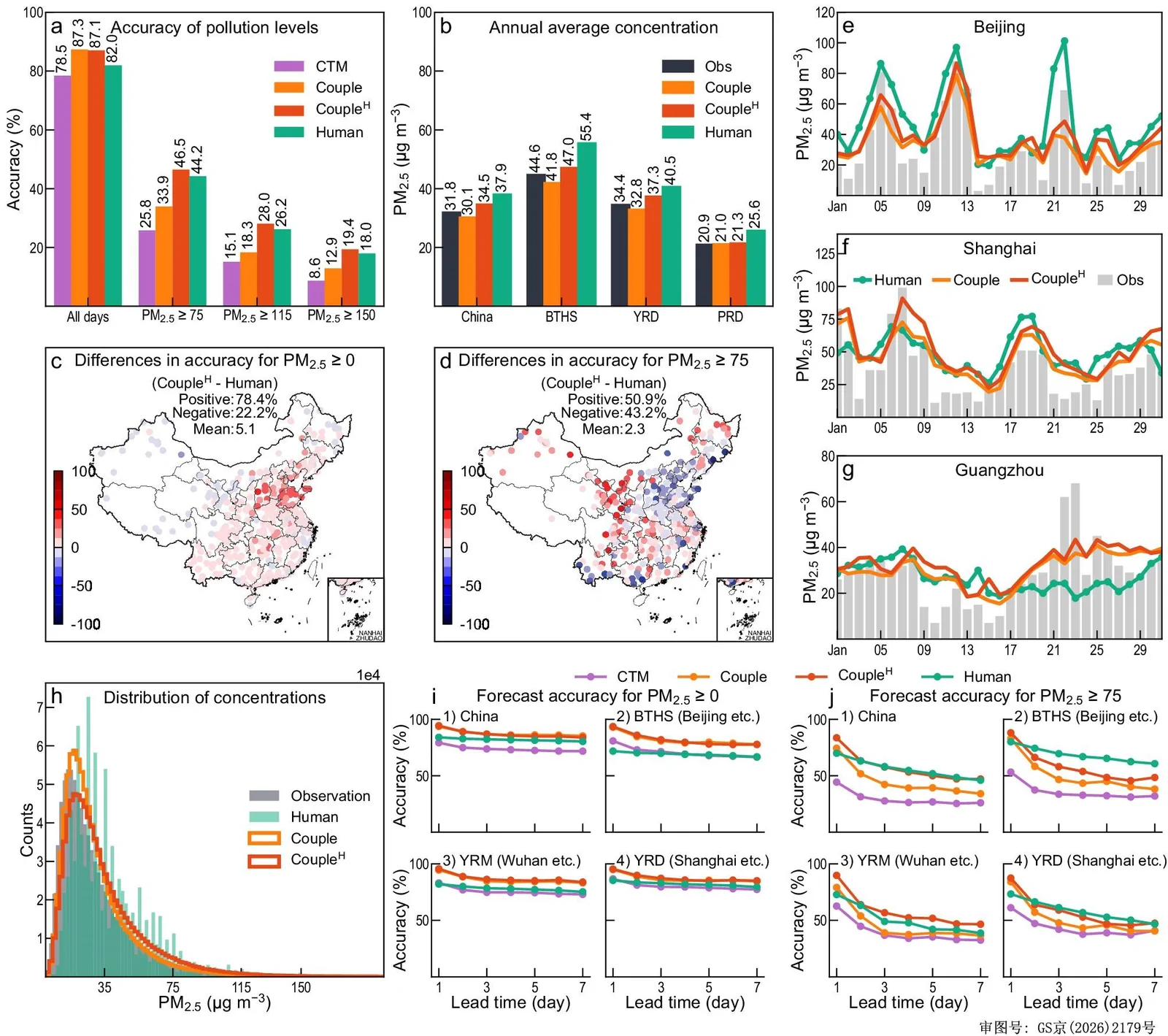
Model fine-tuning to improve PM_2.5_ forecast accuracy for polluted days. (a) Forecast accuracy stratified by PM_2.5_ concentration thresholds. ‘Couple’ denotes baseline ProNet model, while ‘Couple^H^’ represents the fine-tuned variant, which slightly sacrifices overall accuracy to improve performance for high-pollution events. Couple^H^ denotes the coupled model trained with a pollution-aware loss that upweights polluted days, whereas ProNet uses the standard loss. Severe pollution episodes are defined as days with daily-mean PM_2.5_ > 75 μg m^−3^. This ablation quantifies the polluted-day trade-off and shows how the pollution-aware loss improves forecast accuracy under polluted conditions. (b) Annual average concentrations of observed and forecasted PM_2.5_, showing that human forecasts are significantly overestimated, particularly in the BTHS region. (c, d) Accuracy comparisons for all days (c, PM_2.5_ > 0 μg m^−3^) and polluted days (d, PM_2.5_ > 75 μg m^−3^). (e–g) Forecasts for Beijing (e), Shanghai (f) and Guangzhou (g) in January 2023. (h) Density distributions of forecasted versus observed PM_2.5_ concentrations, showing that both human and Couple^H^ forecasts are more frequently distributed at higher concentrations. (i, j) Forecast accuracy for all days (i, PM_2.5_ > 0 μg m^−3^) and polluted days (j, PM_2.5_ > 75 μg m^−3^) by station.

Ozone forecasting shows comparable progress (Figs S6 and S8), demonstrating lower RMSE in 94.7% of cities and a 19.6% reduction in average RMSE from 29.6 to 23.8 μg m^−3^. Compared to PM_2.5_, O_3_ exhibits a shorter chemical lifetime (several hours versus days), resulting in reduced sensitivity to ICs and consequently smaller improvements in 1-day forecasts. Nevertheless, the national average accuracy is increased from 85.5% to 88.5% with spatially heterogeneous gains: 3.1% in BTHS, 2.5% in YRD and 4.0% in PRD.

Although ProNet exhibits lower RMSE across most days and prediction windows (Fig. 5d), two limitations emerge from exceptional events. First, the lack of social activity data integration makes it fail to capture pollution spikes associated with major festivals featuring concentrated firework emissions—notably during the Chinese New Year (22 January 2023) and Lantern Festival (5 February 2023) celebrations. Second, the discrepancies between training and validation periods lead to reduced model performance during dust events (20–23 March 2023 and 4–20 April 2023), because the training year (2022) exhibited below-average dust activity [45] while spring 2023 experienced 2.8 times more dust events than the 10-year climatological mean [46].

## DISCUSSION

Air quality forecasting is critical for the design of pollution control strategies to mitigate air pollution, which causes millions of premature deaths every year [25]. Our ProNet approach leverages the interpretability of process-driven models while compensating for their deficiencies through deep neural networks. Compared with purely AI-based models, ProNet more effectively assimilates observations, predicts pollution variations and adapts to extreme events beyond training data. Compared with human forecasting that similarly utilizes CTM outputs, ProNet demonstrates enhanced capacity to learn from large datasets and capture complex spatiotemporal and interspecies patterns. These advantages enable ProNet to address two critical challenges. First, limited training data availability: This study utilizes 1 year of training data because CTM undergoes continuous mechanistic refinement and observational networks expand rapidly. This dynamic evolution renders long-term training datasets potentially suboptimal for identifying systematic CTM uncertainties. Second, substantial discrepancies between training and testing datasets: The training period (2022) coincided with China’s COVID-19 lockdowns, whereas the testing period (post-8 January 2023) followed lockdown lifting and experienced most sand storms in recent years. Notwithstanding these challenges, ProNet outperformed human operational forecasts, achieving RMSE reductions of 18.4% and an accuracy increase of 6.1%. The framework also provided interpretable insights revealing uncertainties in current PM_2.5_ formation mechanisms and identifying the spatial and temporal sources of CTM forecasting errors.

This study acknowledges three principal limitations that warrant further investigation. First, similar to deep-learning-based forecasting systems, ProNet’s spatial resolution remains constrained by GPU memory limitations. Future research efforts could focus on developing a dynamic downscaling module to enhance urban-scale pollution simulations. Second, while the ensemble optimal interpolation method was selected for its computational efficiency in data assimilation, more sophisticated approaches such as the ensemble Kalman filter (EnKF) or four-dimensional variational (4D-Var) assimilation could be implemented to optimize error covariance estimation and improve the physical consistency of assimilated datasets. Third, although our hybrid framework demonstrates superior relative mass-variation consistency compared to purely AI-driven models, residual imbalances persist. Subsequent developments could integrate rigorous mass-balance constraints through physics-informed neural network architectures to ensure strict mass balance for seasonal forecasting.

This study establishes a novel coupling framework between process-driven models and deep learning networks, specifically validated through its implementation in the CTM (NAQPMS) for air quality forecasting. Importantly, this framework is fundamentally model-agnostic and generalizable beyond NAQPMS; since it learns the error residuals of physical operators rather than relying on model-specific architectures, it can be seamlessly migrated to other Eulerian grid-based CTMs (such as CMAQ or CAMx) simply by training the correctors on their respective baseline forecast and assimilation fields. While successful implementation of this framework demands both process-driven modeling expertise and rigorous analysis, key methodological innovations exhibit broader applicability across Earth system components. First, integrating data assimilation with process-driven modules generates enriched training datasets for deep learning components, facilitating submodule pattern recognition even under sparse observational constraints. Second, our ensemble forecasting architecture incorporates dynamic weight adjustment mechanisms that effectively mitigate bias accumulation during iterative cycles. Third, coupling architectures that learn process-driven model uncertainties through data-driven networks, rather than attempting full substitution, create interpretable frameworks for the diagnosis of mechanistic deficiencies. Collectively, these methodological advances offer transformative potential for next-generation Earth system modeling, significantly enhancing both predictive accuracy and diagnostic capabilities of critical processes. Our current understanding of the Earth system remains very limited, primarily due to the accuracy of existing models. By applying our framework, it is possible to both ensure model accuracy and deepen our understanding of the Earth system and its internal feedback processes. This approach can also improve our knowledge of future disaster evolution, thereby greatly enhancing humanity’s capacity to respond to and adapt to the challenges posed by global change.

## MATERIALS AND METHODS

### Datasets

The training dataset contains 371 daily CTM forecasts from 26 December 2021 to 31 December 2022, each with dimensions of 464 (longitude) × 300 (latitude) × 169 (h) and 30 variables (Table S1). The testing dataset similarly contains 371 forecasts from 26 December 2022 to 31 December 2023. Data before 1 January 2023 from overlapping forecasts were used for training, while 2023 data were held for evaluation.

Surface observations were obtained from the China National Environmental Monitoring Center. After quality control, data from 7036 sites (Fig. S2) were used for model training, with a subset of 1577 national-control sites utilized for evaluation.

### Governing equations of the ProNet

ProNet builds upon CTM [13], whose governing equation is given by


(1) \begin{eqnarray*}
c_{t + 1}^{\mathrm{b}} &=& c_t^{\mathrm{b}} + \Big( \nabla \cdot \left( {{K}^{\mathrm{b}}\nabla c_t^{\mathrm{b}}} \right) - \nabla \cdot \left( {{\boldsymbol{v}}_t^{\mathrm{b}}c_t^{\mathrm{b}}} \right) + E_t^{\mathrm{b}}\\
&&+ {R}^{\mathrm{b}}\left( {c_t^{\mathrm{b}},\mathbb{C}_t^{\mathrm{b}}} \right) + {D}^{\mathrm{b}}\left( {c_t^{\mathrm{b}}} \right) \Big)\Delta t,
\end{eqnarray*}

where ${c}^{\mathrm{b}}$ and ${\mathbb{C}}^{\mathrm{b}}$ denote the concentrations of the target and other relevant chemical species, respectively. ${K}^{\mathrm{b}}$ represents the eddy diffusivity, ${{\boldsymbol{v}}}^{\mathrm{b}}$ is the wind vector and ${E}^{\mathrm{b}}$, ${R}^{\mathrm{b}}$ and ${D}^{\mathrm{b}}$ refer to the rates of emission, reaction and deposition processes, respectively. The superscript ‘b’ indicates baseline forecasts generated by the CTMs.

As outlined in Section S1, although atmospheric transport mechanisms are comparatively well understood, substantial uncertainties remain in modeling emissions, reactions and deposition processes. Assuming that the true concentrations follow an analogous form to Equation (1), we derive the governing equation of ETM as


(2) \begin{eqnarray*}
{e}_{t + 1} &=& {e}_t + \nabla \cdot \left( {{K}^{\mathrm{b}}\nabla {e}_t} \right)\Delta t - \\
&&\nabla\cdot \left( {{\boldsymbol{v}}_t^{\mathrm{b}}{e}_t} \right)\Delta t + {\mathrm{\Psi }}\Delta t,
\end{eqnarray*}

where $e = c - {c}^{\mathrm{*}}$ defines the CTM forecast error, where ‘*’ signifies the true value. The term ${\mathrm{\Psi }}$ aggregates errors introduced by uncertainties in emissions, chemical reactions, deposition and transport over a single model time step:


(3) \begin{eqnarray*}
{\mathrm{\Psi }} &=& E_t^{\mathrm{b}} + {R}^{\mathrm{b}}\left( {c_t^{\mathrm{b}},\mathbb{C}_t^{\mathrm{b}}} \right) + {D}^{\mathrm{b}}\left( {c_t^{\mathrm{b}}} \right) \\
&&- E_t^* - {R}^*\left( {c_t^*,\mathbb{C}_t^*} \right) - {D}^*\left( {c_t^*} \right) + \varepsilon ,
\end{eqnarray*}

where *ɛ * captures additional discrepancies due to imperfections in representing transport dynamics.

From Equation (2), it follows that CTM forecast errors themselves undergo advection and diffusion. Consequently, the total error at each step can be conceptually partitioned into two components: the transported error inherited from previous time steps, and the uncertainty error originating from process-level inaccuracies during the current interval.

### The AI correctors in ProNet

To correct the systematic forecasting errors of CTMs, ProNet incorporates two neural corrector modules within the ETM. The emission-deposition corrector employs a modified dual-encoder architecture comprising temporal and convolutional space modules with roughly 20 million parameters to utilize historical errors and meteorological inputs to correct general spatiotemporal uncertainties. The chemical-reaction corrector, featuring a fully connected network devoid of convolutional units with three 1024-neuron hidden layers and ∼2 million parameters, processes multi-species concentrations to correct PM_2.5_ errors from chemical parameterizations. Input variables for emission-deposition corrector include temperature, relative humidity, wind speed and divergence, u- and v-wind components, terrain elevation, land type, planetary boundary layer height, target species concentrations from CTM and changes of concentration beside transportation. Additionally, the historical space encoder incorporates CTM forecast errors and target uncertainty errors from the preceding 7 days. Variables associated with chemical reactions, described in Table S1, are selected as predictors. These include temperature, relative humidity and changes of concentrations beside transportation for 18 chemical species.

These correctors are trained simultaneously using the target uncertainty errors isolated by the physical transport module, with an additional L1 penalty of 0.05 applied to the chemical module to distinguish reaction-specific errors. Detailed specifications of network architectures, layers, parameter counts and training settings are provided in Section S2.

### Training of AI correctors in ProNet

During data preprocessing, baseline forecasts were generated using conventional CTM simulations without data assimilation; observed data were then assimilated to produce analyzed concentrations. The correctors took as input the hourly concentration changes (excluding transport) for each species, with the baseline-analysis differences defining the target uncertainty errors (Fig. 1e).

We jointly trained the emission-deposition and chemical-reaction correctors inside the ETM using CTM outputs, transport-free concentration changes and corresponding errors. The correctors are optimized using a spatially weighted loss function that evaluates errors exclusively in grid cells with valid station observations. To suppress error accumulation during continuous forecasting, we construct a 10-member ensemble using corrector parameters saved at training epochs 291–300. The minor parameter variations across these epochs act as perturbations that counterbalance individual biases. The final ensemble corrector, containing about 18 million parameters across convolutional space and temporal modules, is trained to merge these forecasts using a standard MSE loss for the base model, and a pollution-weighted loss for the high-pollution variant Couple^H^. In the operational phase, the full framework utilizes an ensemble approach totaling ∼240 million parameters. All models ran on an A800 GPU and were optimized with Adam, using learning rates of 2 × 10^−^^3^, 5 × 10^−^^5^ and 2 × 10^−^^4^ for the emission-deposition, chemical-reaction and ensemble correctors, respectively. Each 7-day forecast is treated as a batch for the chemical-reaction corrector and a sample for other correctors. While conventional CTMs and data assimilation steps consume vast computational resources, our AI correctors are highly efficient. The entire multi-epoch training requires 92 h utilizing one A800 GPU and ten 56-core CPU nodes, whereas executing a single multi-day operational forecast takes merely 92 s beside running CTM and data assimilation. More details are provided in Sections S2 and S7.

## Supplementary Material

nwag570_Supplemental_Files

## Data Availability

The complete dataset generated and analyzed in this study totals ∼13.9 TB (1.2 TB training data, 1.2 TB testing data and 11.5 TB model outputs). Due to storage constraints, the full datasets are available from the corresponding author upon reasonable request. However, to facilitate reproducibility, a 34 GB demonstration subset is publicly available (https://ug.link/couplegroup/filemgr/share-download/?id=f4feb4f9d68745dea50cf4898a4156b0). This subset includes all trained model parameters (∼2.6 GB) and complete input data for a single day, which is sufficient to independently run the model and reproduce the forecast case study in Fig. 3. All source codes associated with this study were written in the Julia programming language and publicly accessible through the GitHub repository at https://github.com/wuhuangjianCN/ProNet.
