# Supplementary material for: Fusing physics and AI enhances accuracy and diagnostic capabilities in air quality forecasting
Source: Natl Sci Rev. 2026 Sep 10;13(19):nwag570. doi: 10.1093/nsr/nwag570 (PMC13678820; doi:10.1093/nsr/nwag570)
Supplement: Supplementary file 1 [file nwag570_supplemental_files.zip › SI-ok.docx]

***Supplementary Information for***

Fusing Physics and AI Enhances Accuracy

and Diagnostic Capabilities in Air Quality Forecasting

Huangjian Wu^1,2^, Zifa Wang^*,1,2,3^, Yuanhang Zhang^4^, Wei Lin^5^, Xinwei Li^1^, Meng Gao^6^, Hao Sun^7^, Dawei Zhang^8^, Wei Wang^8^, Xiao Tang^1,2^, Jiang Zhu^1,3^, Yu Song^4^, Chaoqun Ma^9^, Pingqing Fu^10^, Hang Su^1^, Yafang Cheng^9^ & Gregory R. Carmichael^11^

^1^ State Key Laboratory of Atmospheric Environment and Extreme Meteorology, Institute of Atmospheric Physics, Chinese Academy of Sciences, Beijing 100029, China

^2^ Earth System Numerical Simulation Science Center, Institute of Atmospheric Physics, Chinese Academy of Sciences, Beijing 100029, China

^3^ College of Earth and Planetary Sciences, University of Chinese Academy of Sciences, Beijing, 100049, China

^4^ College of Environmental Sciences and Engineering, Peking University, Beijing 100871, China

^5^ School of Mathematical Sciences and Center for Statistical Science, Peking University, Beijing 100871, China

^6^ Department of Geography, Hong Kong Baptist University, Hong Kong SAR 999077, China

^7^ Gaoling School of Artificial Intelligence, Renmin University of China, Beijing 100872, China

^8^ China National Environmental Monitoring Center, Beijing 100012, China

^9^ Aerosol Chemistry Department, Max Planck Institute for Chemistry, 55128 Mainz, Germany

^10^ Institute of Surface-Earth System Science, Tianjin University, Tianjin 300072, China

^11^ Center for Global and Regional Environmental Research, University of Iowa, Iowa City, IA 52242, USA

* Corresponding author. E-mail: zifawang@mail.iap.ac.cn

This document includes Supplementary Section 1–14, Tables S1 to S4, and Figure S1 to S21.

# Supplementary Section 1: Uncertainties in air quality forecasts

Operational air quality forecasting predominantly relies on CTM [1], which simulates the transportation, emission, chemical reaction, and deposition processes (Fig. 1). A generic form of the governing equation is given by

$c_{t+1}^{b}=c_{t}^{b}+\left( \nabla\cdot\left( K^{b}\nabla c_{t}^{b} \right)-\nabla\cdot\left( \boldsymbol{v}_{t}^{b}c_{t}^{b} \right)+E_{t}^{b}+R^{b}\left( c_{t}^{b},\mathbb{C}_{t}^{b} \right)+D^{b}\left( c_{t}^{b} \right) \right)\Delta t$	(1)

where $c^{b}$ and $\mathbb{C}^{b}$ represent the concentrations of the target and other relevant chemical species, respectively. $K^{b}$ denotes the eddy diffusivity, and $\boldsymbol{v}^{b}$ is the wind vector. $E^{b}$, $R^{b}$, and $D^{b}$ are the rate of emission, reaction, and deposition processes, respectively. The superscript "b" denotes the baseline forecasts obtained from CTMs.

Emissions $E_{t}^{f}$ constitute a major source of uncertainty, primarily due to incomplete knowledge of anthropogenic activities and source-specific contributions. These emissions are typically quantified using activity-dependent emission factors derived from source-specific measurements [2]. Temporal variations are modeled through statistical proxies (e.g., Eurostat, ENTSO-E, UN monthly bulletin [3]), while spatial distributions are resolved using geographic indicators including population density, transportation networks, and land use data. Consequently, emission inventories are subject to considerable uncertainties, exhibiting pronounced spatiotemporal variability that can surpass 100% for specific species.

Reaction $R^{f}\left( c_{t}^{f},\mathbb{C}_{t}^{f} \right)$ represents the sole process governing direct interspecies interactions, simulating gas-phase, aqueous-phase, and homogeneous reactions across air, droplets, and particulate phases. To mitigate computational demands, these reactions are simplified by aggregating organic species into fewer components, assuming equilibrium concentrations for rapid reactions, and discretizing particle sizes into limited modes [4]. Such approximations, coupled with parameter uncertainties, may introduce cross-species correlations in CTM forecast errors.

Deposition $D^{f}\left( c_{t}^{f} \right)$ governs the removal of trace gases and particles from the atmosphere [5]. This process depends on variables that are difficult to constrain accurately, including the physicochemical properties of depositing species, land-surface characteristics (e.g., roughness, vegetation cover), hydrometeor properties (cloud/fog droplets, rain, snow), and atmospheric turbulence intensity.

Transportation $\nabla\cdot\left( K^{f}\nabla c_{t}^{f} \right)-\nabla\cdot\left( \mathbf{v}_{t}^{f}c_{t}^{f} \right)$, which encompasses advection and diffusion, governs the movement of trace gases and particles in the atmosphere. Given accurate wind vectors, errors arising from this process become negligible, as diffusion is orders of magnitude slower than advection [6], and advection itself is well-characterized with minimal uncertainty [7].

Uncertainties in these processes introduce errors at each time step of the CTM forecast. These errors are subsequently transported and accumulated, ultimately contributing to the total forecast error. As shown in Figs. S11 and S12, the error transported from the previous time step (Transported Error) constitutes the dominant fraction of total forecast errors, governing their spatiotemporal evolution at short timescales. In contrast, the Uncertainty Error, unaffected by temporal or spatial mixing processes, offers direct insights into system uncertainties associated with emission, reaction, and deposition processes. While process-driven models can precisely quantify the Transported Error by its definition, they remain inherently limited in capturing the Uncertainty Error, which reflects fundamental gaps in our current scientific understanding.

# Supplementary Section 2: The physics and AI components

## Supplementary Section 2.1: The physics based Chemical Transport Model

The Chemical Transport Model (CTM) we employed in ProNet is the Nested Air Quality Prediction Modeling System (NAQPMS) [8], which is developed by the Institute of Atmospheric Physics, Chinese Academy of Sciences,. NAQPMS utilizes a terrain-following coordinate system for transport processes. The transport module incorporates a diffusion scheme by Byun and Dennis [9] and an advection scheme by Walcek and Aleksic [7], which is mass-conservative, peak-preserving, and mixing-ratio-bounded. The reaction process comprises four modules: (1) the Carbon Bond Mechanism Z (CBM-Z) [10], which calculates 133 reactions among 53 gas-phase species; (2) the aerosol thermodynamic model ISORROPIA v1.7 [4], simulating inorganic aerosols in an ammonia–sulfate–nitrate–chloride–sodium–water system; (3) a module for secondary organic aerosol formation from anthropogenic [11] and biogenic [12] precursors; and (4) a module simulating 28 heterogeneous reactions involving 14 species (e.g., dust, sea salt, sulfate, and black carbon) [13]. Deposition processes include a dry deposition scheme [14] and a wet deposition scheme [15]. Emission inventories integrate multiple sources: anthropogenic emissions (MIX inventory [2]) , biogenic and biomass burning emissions [16, 17] and dust/sea salt emissions [18, 19].

Meteorological input fields were derived from the Weather Research and Forecasting (WRF) model [20], utilizing initial and boundary conditions obtained from the National Centers for Environmental Prediction’s Global Forecast System (GFS). The domain configurations for both WRF and NAQPMS are illustrated in SI Fig. S2. The outer domain encompasses East Asia with a 45-km horizontal resolution (228 × 165 grid points), while the inner domain covers the Chinese mainland with a 15-km horizontal resolution (464 × 300 grid points). Both domains comprise 20 vertical layers, including 8 layers within the lower 2 km of the atmosphere.

## Supplementary Section 2.2: The AI Correctors

As illustrated in Fig. 1, the ProNet comprises three correctors. The Emission-Deposition Corrector and Chemical-Reaction Corrector—designed using a video prediction network (SimVP) and a fully connected network, respectively—mitigate Uncertainty Errors in the chemical transport model (CTM). The Ensemble Corrector, also based on SimVP, reduces bias accumulation during forecasting.

While the current framework isolates chemical reactions from the combined emission and deposition processes, this categorization is simplified from a strict physical perspective. Ideally, distinct mechanisms such as dry deposition, wet deposition, and subgrid-scale turbulent diffusion should be explicitly decoupled. However, process-level observations are currently extremely scarce, making it nearly impossible to obtain sufficient specific target data to independently train these highly specialized sub-modules. In our approach, we sequentially assimilated observations to construct continuous concentration fields, isolated physical transport errors, and separated the remaining uncertainties into two grouped correctors. Because each mathematical decomposition step inherently introduces some level of data noise, further subdividing the correctors without robust, specific observational constraints would make it exceedingly difficult to effectively decouple the highly entangled physical signals during training. Such over-segmentation could lead to spurious compensations between the sub-networks, potentially failing to capture the intended process uncertainty features and ultimately degrading both forecasting performance and physical interpretability. Therefore, adhering to the principle of parsimony, we maintained the current grouped design. As high-quality multi-species observational data increase and decadal process-level training datasets are established in the future, explicitly separating specific deposition mechanisms and turbulent diffusion processes will be a crucial next step to further enhance the diagnostic resolution of the framework.

**Emission-Deposition Corrector.** The Emission-Deposition Corrector is designed to learn the spatiotemporal patterns of Uncertainty Error. This task conceptually resembles video prediction, prompting the adoption of SimVP, a convolutional neural network (CNN)-based video prediction model. Notably, SimVP has demonstrated superior performance over recurrent or adversarial training-based models across multiple benchmark datasets. The model employs a streamlined encoder-translator-decoder architecture that integrates three core components: (i) spatial feature extraction through convolutional blocks, (ii) temporal dynamics modeling via inception-based translation modules, and (iii) future frame reconstruction using transposed convolutions.

Building upon the SimVP framework, we introduced three key architectural improvements for air quality forecasting (Fig. 1b). First, the proposed corrector employs dual encoders (rather than a single encoder) to process future and past information separately, enabling simultaneous estimation of emission and deposition uncertainties based on future meteorological conditions and historical emission patterns. Second, we implemented deeper encoder-decoder architectures to handle higher spatial resolutions (464×300 pixels). Third, expansion of input channel from 3 to 13 in the forecast-space encoder, including temperature, relative humidity, wind speed and divergence, u- and v-wind components, terrain elevation, land type, planetary boundary layer height, target species concentrations from CTM, and changes of concentration beside transportation. Additionally, the historical space encoder incorporates CTM forecast errors and target uncertainty errors from the preceding seven days, allowing the Emission-Deposition Corrector to capture dynamically evolving emission patterns.

**Chemical-Reaction Corrector.** The Chemical-Reaction Corrector is designed to capture cross-species patterns in Uncertainty Error arising from chemical reaction processes. Variables associated with chemical reactions, described in Table S1, are selected as predictors. These include temperature, relative humidity, and changes of concentrations beside transportation for 18 chemical species. Given the absence of spatial and temporal interactions in chemical reactions, the Chemical-Reaction Corrector employs a fully connected neural network architecture devoid of convolutional or recurrent units. As depicted in Fig. 1d, the Corrector comprises an output layer and three stacked blocks composed of fully connected layers with hyperbolic tangent activation functions and dropout regularization. The use of smooth activations enables this architecture to learn complex nonlinear relationships, making it one of the most promising approaches in nonparametric regression [21].

**Ensemble Corrector**. The Ensemble Corrector is designed to mitigate systematic bias accumulation during forecasting. It resembles the Emission-Deposition Corrector but incorporates one encoder and two decoders (Fig. 1c). The first decoder employs a softmax layer at its output to generate normalized ensemble weights that sum to unity, while the second decoder directly corrects ensemble biases. In addition to the 13 predictors identical to those in the forecast space encoder of the Emission-Deposition Corrector, this corrector integrates 10 ensemble forecasts generated by a CTM and ten ETM. The target output of this corrector is the assimilated data. The primary rationale behind this design is to mitigate the error accumulation caused by inevitable small perturbation biases during continuous AI model forecasting. Due to the inherent stochasticity in the neural network training process (e.g., mini-batch data sampling), the AI correctors at different epochs converge to slightly different local minima. Consequently, the AI module parameters differ slightly across these final epochs. This variation essentially acts as a data-driven perturbation. By selecting multiple epochs, some AI modules will naturally produce slightly higher estimates while others produce lower ones. When these ten members are combined and processed through the proposed AI Ensemble Corrector module, the diverse biases counterbalance each other, thereby effectively suppressing the error accumulation during the forecast integration.

**Pure-AI Model**. In contrast to the ProNet, the purely AI-based model removes all process-based modules and CTM inputs. As illustrated in Fig. S1, the architecture consists of two modules analogous to ProNet’s Emission-Deposition Corrector and Ensemble Corrector. The Member Predictor module integrates a forecast space encoder with nine input variables: planetary boundary layer height, temperature, relative humidity, wind speed, wind divergence, u- and v-wind components, terrain elevation, and land type. The Ensemble Predictor module mirrors the Ensemble Corrector but excludes all CTM-derived predictors. Both predictors are trained using assimilated data as targets.

**Loss function.** The ProNet approach incorporates two primary loss functions. As components of the ETM architecture, the Emission-Deposition Corrector and Chemical-Reaction Corrector are jointly optimized to predict Uncertainty Errors. The combined loss function $\mathcal{L}^{u}$ is formulated as:

$$\mathcal{L}^{u}=\sum_{i=1}^{N} \omega_{i}(\left| y_{i}^{E}+y_{i}^{R}-y_{i}^{0} \right|+\left| y_{i}^{R}-y_{i}^{0} \right|+\left| y_{i}^{E}-y_{i}^{0} \right|+\alpha\left| y_{i}^{R} \right|)/\sum_{i=1}^{N} \omega_{i}$$

where $y_{i}^{0}$ denotes the target Uncertainty Error at grid cell $i$, $N$ total number of grids, and $y_{i}^{E}$ and $y_{i}^{R}$ represent outputs from the Emission-Deposition Corrector and Chemical-Reaction Corrector, respectively. The weighting coefficient $\omega_{i}$ is set to 1 for grid cells within the characteristic length scale of data assimilation (where observations are available nearby) and 0 otherwise, thereby restricting the evaluation to observed regions. An additional L1 regularization term $\alpha\left| y_{i}^{R} \right|$, with $\alpha$ set to 0.05, is applied to the Chemical-Reaction Corrector to prioritize that residual errors captured by the Chemical-Reaction Corrector more distinctly reflect reaction-specific uncertainties.

For the baseline Ensemble Corrector, the loss function $\mathcal{L}^{e}$ is formulated as:

$$\mathcal{L}^{e}=\sum_{i=1}^{N} \omega_{i}{(y_{i}^{\mathrm{En}}-c_{i}^{a})}^{2}/\sum_{i=1}^{N} \omega_{i}$$

where the final forecast $y_{i}^{\mathrm{En}}=y_{i}^{b}+\sum_{j}^{M} (y_{i,j}^{w}c_{i,j}^{m})$, $y_{i,j}^{w}$ and $y_{i}^{b}$ denote the space decoder outputs for ensemble weights and bias, respectively; $c_{i}^{a}$ is the assimilated pollutant concentration, $c_{i,j}^{m}$ the forecast concentrations of ensemble members, and M the number of ensemble members.

For the fine-tuning Ensemble Corrector designed to improve forecasting accuracy in highly polluted days, the loss function $\mathcal{L}^{H}$ is formulated as:

$$\mathcal{L}^{H}=\sum_{i=1}^{N} \omega_{i}P_{i}{(y_{i}^{\mathrm{En}}-c_{i}^{a})}^{2}/\sum_{i=1}^{N} \omega_{i}$$

where $P_{i}$ is the pollution level of $c_{i}^{a}$, ranging from 1 to 6.

## Supplementary Section 2.3: The transport module

The transport module is implemented both in CTM and ETM. It represents the sole process governing spatial interactions in the CTM, dominating the short-term spatiotemporal evolution of forecasts and their associated errors (Supplementary Figs. S5-6). This process integrates information from disparate regions and temporal steps during forecasting, complicating the analysis of other processes. Nevertheless, its mechanistic basis is well established and can be formalized as follows:

$\frac{\partial c}{\partial t}=\nabla\cdot\left( K\nabla c \right)-\nabla\cdot\left( \boldsymbol{v}c \right)$

where $c$ denotes species concentration, $K$ the eddy diffusivity, and $\boldsymbol{v}$ the velocity field. The operator $\nabla$ represents gradient while $\nabla\cdot$ indicates divergence. The first term $\nabla\cdot\left( K\nabla c \right)$ governs molecular diffusion, a process typically orders of magnitude slower than advection in regional air quality applications [6]. We compute diffusive fluxes using a turbulence scheme [22] that parameterizes $K^{f}$ through wind shear and grid-scale dependencies. The advective term $(-\nabla\cdot\left( \mathbf{v}c \right))$ is solved using a rigorously mass-conservative transport scheme that preserves extrema and maintains positive definiteness [7]. The scheme incorporates targeted modifications at local extrema and utilizes piecewise dual-linear reconstructions to effectively preserve peak values and minimize numerical diffusion. The complete numerical implementation of the advection scheme is described below.

The governing equation incorporates the mixing ratio through the following formulation:

$\frac{\partial\left( \rho Q \right)}{\partial t}=-\nabla\cdot\left( \boldsymbol{v}\rho Q \right)$

where $\rho$ denotes the fluid density and $Q=c/\rho$ represents the mixing ratio. "In multidimensional implementations, the scheme employs dimension-splitting to sequentially update $Q$ across all spatial dimensions, with the grid-cell $(i, j)$ mixing ratio discretized as:

$Q_{i}^{t+\Delta t}=\left( Q_{i}^{t}D_{0}-\frac{F_{i+1/2}}{\Delta x_{i}}+\frac{F_{i-1/2}}{\Delta x_{i}} \right)/{D_{1}}$

$Q_{j}^{t+\Delta t}=\left( Q_{j}^{t}D_{1}-\frac{F_{j+1/2}}{\Delta y_{j}}+\frac{F_{j-1/2}}{\Delta y_{j}} \right)/{D_{2}}$

where $\Delta x_{i}$ and $\Delta y_{j}$ denote the grid spacings in the x- and y-directions, respectively, $\Delta t$ represents the time step size, $D_{k}$, $k=0, 1, 2$, are dimensionally dependent fluid densities, and $F_{i\pm1/2}$ and $F_{j\pm1/2}$ correspond to the normal-directed tracer fluxes at cell interfaces.

Mass conservation is enforced through the density update scheme:

$D_{0}=\rho_{0i}$

$D_{1}=D_{0}-[\left( \rho_{0}v_{1} \right)_{i+1/2}-\left( \rho_{0}v_{1} \right)_{i-1/2}]\Delta t/\Delta x_{i}$

$D_{2}=D_{1}-[\left( \rho_{0}v_{2} \right)_{j+1/2}-\left( \rho_{0}v_{2} \right)_{j-1/2}]\Delta t/\Delta y_{j}$

where $\rho_{0i}$ denotes initial fluid densities, while $\left( \rho_{0}v_{1} \right)_{i\pm1/2}$ and $\left( \rho_{0}v_{2} \right)_{j\pm1/2}$ represent the fluid fluxes across cell faces. The corresponding tracer fluxes Ψ are then computed as:

$F_{i+1/2}={(\rho_{0}v_{1})}_{i+1/2}\Delta tQ_{i}^{f}$

$F_{j+1/2}={(\rho_{0}v_{2})}_{j+1/2}\Delta tQ_{j}^{f}$

where $Q_{i}^{f}$ and $Q_{j}^{f}$ are the outflowing mixing ratios. Complete derivation of the flux calculations and $Q$-limiting procedures can be found in Walcek and Aleksic [7].

# Supplementary Section 3: The data assimilation method

We employed the Ensemble Optimal Interpolation (EnOI) [23] method as our data assimilation approach to generate assimilated datasets specifically tailored for deep neural network training. Unlike dynamic ensemble methods such as the Ensemble Kalman Filter (EnKF), EnOI constructs ensembles directly from historical or climatological data, eliminating the need for iterative model runs. This strategy substantially reduces computational expenditure while preserving adequate predictive accuracy. The analysis state $\boldsymbol{c}^{a}$ is computed as:

$\boldsymbol{c}^{a}=\boldsymbol{c}^{b}+\boldsymbol{P}\boldsymbol{H}^{T}\left( \boldsymbol{HP}\boldsymbol{H}^{T}+\boldsymbol{R} \right)^{-1}(\boldsymbol{y}-\boldsymbol{H}\boldsymbol{c}^{b})$

where $\boldsymbol{c}^{b}$ denotes the background field (CTM forecasts), respectively. $\boldsymbol{y}$ is the observation vector, $\boldsymbol{H}$ represents the observation operator, while $\boldsymbol{B}$ and $\boldsymbol{R}$ signify the background and observation error covariance matrices, respectively.

Following a common practice in data assimilation, we assume observational independence, rendering the observation error covariance matrix $\boldsymbol{R}$ diagonal. The background error covariance matrix $\boldsymbol{P}$ is calculated by:

$\boldsymbol{P}={\boldsymbol{\rho}○\boldsymbol{A}}^{b}{\boldsymbol{A}^{b}}^{T}/(m-1),$

where $\boldsymbol{\rho}$ represents the localization matrix derived from a covariance localization scheme [24], implemented to mitigate sampling errors. Matrix elements are determined through the Gaspari-Cohn [25] tapering function, with the localization radius varying between 15 km and 150 km based on the proximity of available observations. The ensemble size m is set to 9 in this study. The forecast ensemble anomalies $\boldsymbol{A}^{b}$ are defined as deviations between the forecast ensemble states $\boldsymbol{X}^{b}$ and their ensemble mean:

$\boldsymbol{A}^{b}=\boldsymbol{X}^{b}(\boldsymbol{I}-\boldsymbol{1/}m),$

where $\boldsymbol{I}$ denotes the identity matrix and $\boldsymbol{1}$ represents a matrix with identical dimensions to $\boldsymbol{I}$, whose elements are all unity. The forecast ensemble members $\boldsymbol{X}^{b}$ are uniformly distributed, centered on the CTM forecasts. The ensemble spread is determined as four times the domain average of CTM predictions plus twice the grid-specific CTM forecast values.

Through this EnOI implementation, we generated assimilated datasets for PM_2.5_ concentrations across all CTM forecasts. Validation results are presented in S9 and S10.

# Supplementary Section 4: Techniques to reveal uncertainties in current PM_2.5_ formation mechanisms

The proposed methodology identifies key uncertainties in PM_2.5_ formation mechanisms through four sequential steps. First, data assimilation is employed to generate spatiotemporally resolved estimates of CTM forecast errors by integrating extensive observational datasets. Second, the transport module decomposes CTM forecast errors into Transported Error and Uncertainty Error components, effectively decoupling uncertainties across temporal and spatial domains. Third, Emission-Deposition and Chemical-Reaction Correctors further partition Uncertainty Error to isolate cross-species errors arising from reaction process uncertainties. Finally, interpretable techniques—including SHapley Additive exPlanations (SHAP) [26] and automatic differentiation [27]—quantify the contribution of each predictor.

For the SHAP analysis in Fig. 2d–f, SHAP was applied to the trained PM_2.5_ uncertainty-error corrector to quantify how each input feature contributes to the learned CTM-error correction. The input features include chemical-component error terms and meteorological variables used by the corrector. SHAP quantifies the contribution of each feature to the model output via the computation of Shapley values, defined as:

$\phi_{i}=\sum_{S\subseteq N\setminus\{i\}} \frac{\left| S \right|!\left( \left| N \right|-\left| S \right|-1 \right)!}{\left| N \right|!}\left[ f_{S\cup\{i\}}\left( x_{S\cup\{i\}} \right)-f_{S}\left( x_{S} \right) \right]$	(1)

where $\phi_{i}$ represents the SHAP value for feature i, and N denotes the full feature set, S denotes the feature subset excluding feature i. SHAP satisfies additivity property:

$f\left( x \right)=E\left[ f\left( x \right) \right]+\sum_{i=1}^{M} \phi_{i}$	(2)

where, $f\left( x \right)$ is the model prediction for a single sample, $E\left[ f\left( x \right) \right]$ denotes the expected predicted value over the background samples, and M is the number of features. Feature importance in Fig. 2d was ranked by the mean absolute SHAP value across all grid–time samples. SHAP values are used here as diagnostic indicators of systematic CTM error-correction patterns.

It is crucial to emphasize that this technique is based on observations of total PM_2.5_ concentrations. Even in the absence of sparse data on specific PM_2.5_ components, our methodology remains capable of identifying potential overestimations in component formation and performing conditional analyses. Furthermore, through data assimilation and the methodological framework outlined above, our approach effectively minimizes confounding effects from transport, emission, and deposition processes.

# Supplementary Section 5: Procedures to calculate forecasting accuracy

In this study, we implement a standardized framework to evaluate the predictive accuracy of air quality models. The framework comprises two sequential steps: first, deriving forecasted Air Quality Index (AQI) values in strict compliance with China’s Technical Regulation on Ambient Air Quality Index (HJ 633-2012); and second, applying accuracy assessment protocols following the Technical Regulation on Urban Ambient Air Quality Index Forecast Evaluation (Interim).

The forecasted AQI is calculated from two key pollutants: PM_2.5_ (based on 24-hour mean concentrations) and O_3_ (determined from daily maximum 8-hour moving averages, obtained from continuous 24-hour monitoring). These concentrations are then transformed into Individual Air Quality Indices (IAQI) via a piecewise linear interpolation method:

$$A^{o}=\frac{A_{i+1}-A_{i}}{C_{i+1}-C_{i}}\left( C^{o}-C_{i} \right)+A_{i}$$

where $C^{o}$ represents the forecasted pollutant concentration. $C_{i}$ and $C_{i+1}$ denote the threshold endpoints for pollutant concentration, while $A_{i}$ and $A_{i+1}$ correspond to the IAQI endpoints (Table S4).

The forecast interval is determined by the forecast IAQI: for IAQI ≤50, the permissible error is ±10 (with a lower limit of 0); for IAQI >50, the permissible error is ±20% (rounded upward). A forecast is deemed accurate if the observed AQI level lies within the air pollution category overlaps with the forecast interval.

The comprehensive accuracy rate is defined as the ratio of accurate forecasts to the total number of forecasts during the evaluation period, calculated as follows:

$$Accuracy Rate \left( \% \right)=\frac{Number of Accurate Forecasts}{Total Forecasts}\times100$$

This approach establishes a standardized and reproducible framework for assessing the performance of air quality predictions.

# Supplementary Section 6: Human-adjusted operational forecasts and alignment protocol

The human benchmark used in this study is the official forecaster-adjusted city-level operational forecast product. The product is issued daily at 17:00 local time (UTC+8) and provides D+1 to D+7 forecasts of daily-mean PM_2.5_ For each lead day, the forecast target is the local daily mean over 00:00–23:00.

To ensure an apples-to-apples comparison, ProNet, CTM, and AI-only model outputs are first aggregated from hourly concentrations to daily means using the arithmetic mean of the 24 hourly values over the same local 00:00–23:00 window for each lead day. All methods are then evaluated on the same city–day–lead samples for which the forecaster-adjusted operational forecast is available. The full evaluation set contains 863,590 city–day–lead entries (365 days × 338 cities × 7 lead days). The human forecast product is missing for 2,422 entries, corresponding to 0.28% of the full set. These entries are excluded from the human-benchmark comparison, resulting in a common evaluation subset of 861,168 paired forecasts. This exclusion has negligible influence on the results, with RMSE values unchanged to one decimal place.

Operational forecasters may use additional information that is not available to ProNet, including local forecasting systems, local emission inventories, real-time observations, meteorological guidance, and accumulated expert knowledge. We therefore frame this comparison as a stringent benchmark against a human-in-the-loop operational product rather than a strictly same-information comparison.

The national air quality forecasting system employs a multi-step expert-guided framework across 339 Chinese cities, where provincial teams (2–8 trained forecasters, scaled by regional pollution and regulatory needs) refine numerical predictions through five integrated procedures: (1) Operational CTMs (NAQPMS, WRF-Chem, CMAQ, CAMx etc.) generate baseline forecasts compliant with HJ 1130-2020, utilizing dynamic emission inventories (MEIC/HTAP-based, sector-time adjusted) and assimilated chemical initial/boundary conditions; (2) Source apportionment via hybrid receptor modeling (PMF-CMB with HYSPLIT trajectories) and sensitivity simulations quantifies local/regional contributions, incorporating empirical adjustments for transient controls or systematic inventory biases; (3) Dispersion potential is assessed through multi-model meteorological ensembles (ECMWF/CMA/GFS) with historical analog corrections for significant forecast discrepancies; (4) Real-time monitoring data—from a hierarchical network fused with satellite fire detections (VIIRS/MODIS) and aerosol profiles (CALIPSO)—identifies unmodeled emission events (e.g., fires, dust), with adjustments via wind and trajectory analysis; (5) Forecasters apply weighted synthesis leveraging regional pollution expertise to produce city-level predictions, which are disseminated through official government platforms (Ministry of Ecology and Environment Weibo for 36 key cities [https://m.weibo.cn/u/6059162597], "Air Quality Publishing" application for all cities [http://qr1.weigongju.org/16604580]) with daily updates.

# Supplementary Section 7: Procedures of the ProNet approach

During the training phase, the Emission-Deposition, Chemical-Reaction, and Ensemble Correctors are optimized via the following workflow:

1) Baseline generation: Conventional CTM simulations with non-assimilated ICs provide baseline forecasts.
2) Data assimilation: Observations are assimilated into the CTM to produce the assimilated states at each time step.
3) Process isolation: Calculate the changes of concentrations in an hour beside transportation for each CTM species. These variables, e.g. ΔPM_2.5_, are served as the inputs for Corrector in ETM, and the changes of baseline forecasting error beside transport are serve as the target Uncertainty Error for model training. By separating the total error into the error transported from the previous hour and the new error generated in the current hour at each hourly step, we prevent the accumulation of errors in the training targets.
4) Train ETM: Jointly train Emission-Deposition and Chemical-Reaction Correctors in ETM using CTM forecasts, changes of concentrations beside transportation and corresponding Uncertainty Error.
5) Ensemble construction: A 10-member ensemble forecast is generated through the integration of a single CTM output with ten distinct ETM simulations, each utilizing parameters obtained from different training epochs.
6) Train Ensemble Corrector: Train Ensemble Corrector with ensemble forecasts as inputs and assimilated concentrations as targets. The Ensemble Corrector is designed to identify and correct systematic bias accumulation during recursive multi-step forecasting.

For operational forecasting, the trained correctors are deployed through a five-stage pipeline:

1) Baseline generation: Execute conventional CTM forecast with non-assimilated ICs.
2) Prepare ICs for ETM: a) Assimilate observations into CTM ICs. b) Compute ETM ICs as the difference between assimilated and non-assimilated CTM ICs.
3) Prepare inputs for Correctors in ETM: Compute changes of concentrations in an hour beside transportation for CTM species as inputs for the ETM correctors.
4) Ensemble generation: Conduct ten ETM forecasts and combine with the CTM outputs to generate ensemble forecasts.
5) Final ensemble correction: The Ensemble Corrector is applied to the ensemble forecasts to produce the final ProNet forecast.

For the above procedures, most time and computational resources are consumed by the CTM and data assimilation. The remaining process-driven models and data-driven networks were run on 1 A800 GPU and 10 CPU nodes (56 cores per node), which took 92 hours for training and only 92 seconds for a forecasting cycle, adding negligible computational overhead to the CTM workflow.

# Supplementary Section 8: Relative Diagnostics for Dynamical Consistency with Process-Based Baselines

To quantitatively evaluate the physical resemblance of the forecasting models, we designed three relative indices to assess relative mass-variation consistency, temporal smoothness, and transport realism. The diagnostic metrics below evaluate the relative physical resemblance of target models against the baseline Chemical Transport Model (CTM) and its physical transport laws, rather than an absolute thermodynamic or mass-conservation standard. In operational CTMs, continuous hourly mass fluxes from emissions and deposition, alongside numerical diffusion, discretization errors, and parameterized sub-grid processes (e.g., turbulent mixing), mean that atmospheric pollutant mass varies dynamically over time. Therefore, these metrics quantify how closely a data-driven or hybrid model adheres to the dynamical core and physical transport governing equations of the underlying process-based CTM.

1. Relative Mass-Variation Consistency (Index for Relative Mass Imbalance) The Index for Mass Imbalance ($I_{t}^{mass}$) evaluates the degree of mass conservation by calculating the ratio between the absolute difference in hourly concentration changes (target model versus CTM) and the average concentration of the CTM within the evaluation region. The formula is defined as:

$$I_{t}^{mass}=\sum_{i} \frac{\left| c_{i,t}^{m}-c_{i,t-1}^{m}-{(c}_{i,t}^{ctm}-c_{i,t-1}^{ctm}) \right|}{\sum_{i} c_{i,t}^{m}}$$

where $c_{i,t}^{m}$ and $c_{i,t}^{ctm}$ represent the simulated concentrations at grid point *i* and time *t* for the evaluated model *m* and the baseline CTM, respectively. A larger $I_{t}^{mass}$ value indicates a greater deviation in mass variation from the physical CTM baseline.

**2.** **Temporal Smoothness (Index for** **Deviation of Temporal Variance)** The temporal smoothness of the forecasts is measured by the second derivative of the concentrations, which captures abrupt temporal fluctuations. The Index for Deviation of Temporal Variance ($I_{t}^{variance}$) is calculated by normalizing the absolute difference between the second derivatives of the evaluated model and the CTM using the average CTM concentration:

$$I_{t}^{variance}=\sum_{i} \frac{\left| c_{i,t+1}^{m}-{2c}_{i,t}^{m}-c_{i,t-1}^{m}) \right|-\left| c_{i,t+1}^{ctm}-{2c}_{i,t}^{ctm}-c_{i,t-1}^{ctm}) \right|}{\sum_{i} c_{i,t}^{m}}$$

where $c_{i,t+1}^{m}-{2c}_{i,t}^{m}-c_{i,t-1}^{m}$ acts as the discrete approximation of the second derivative of concentration with respect to time. A larger $I_{t}^{variance}$ value signifies that the temporal smoothness of the target model deviates more significantly from the CTM, indicating unphysical, abrupt fluctuations.

**3. Transport Realism (Index of Transport Realism)** To evaluate whether the model’s predictions adhere to atmospheric transport dynamics, we formulated the Index of Transport Realism ($I_{t}^{Transport}$). This index calculates the spatial Pearson correlation coefficient between the hourly concentration changes produced by the target model and the expected concentration changes driven purely by the physical transport module. The formula is:

$$I_{t}^{Transport}=\frac{\sum_{i} (\Delta c_{i,t}^{m}-\bar{\Delta c_{i,t}^{m}})(\Delta c_{i,t}^{T}-\bar{\Delta c_{i,t}^{T}})}{\sqrt{\sum_{i} (\Delta c_{i,t}^{m}-\bar{\Delta c_{i,t}^{m}})\sum_{i} (\Delta c_{i,t}^{T}-\bar{\Delta c_{i,t}^{T}})}}$$

where ${\Delta c_{i,t}^{m}=c}_{i,t}^{m}-c_{i,t-1}^{m}$ represents the concentration change produced by the evaluated model *m*. ${\Delta c_{i,t}^{T}=c}_{i,t}^{T}-c_{i,t-1}^{m}$ represents the expected concentration change, where $c_{i,t}^{T}$ is the theoretically transported concentration derived by applying the physical transport module to the model’s previous state ($c_{i,t-1}^{m}$). The overbars ($\bar{\Delta c_{i,t}^{m}}$ and $\bar{\Delta c_{i,t}^{T}}$) denote the spatial means of these changes. A higher $I_{t}^{Transport}$ value (closer to 1.0) indicates that the model’s concentration variations are more consistent with physical transport laws.

# Supplementary Section 9: Spatiotemporal Analysis of Forecast Error Attribution

To trace the origins of forecast errors and isolate local uncertainties from transboundary transport, we employed a spatiotemporal tagging technique within the coupled physical framework. Initial concentration fields and hourly model uncertainty errors originating from distinct geographic source regions and temporal windows were assigned unique numerical tags and explicitly tracked through the process-driven advection-diffusion transport module. For the severe Beijing PM_2.5_ overestimation episode on January 15, 2023, tracking the spatiotemporal error tags revealed that the primary error contributions did not originate locally within Beijing, but were overwhelmingly transported from upwind heavy-industrial regions in Shanxi and Shandong provinces. As demonstrated by the synoptic wind fields and regional pollution evolution in SI Fig. S16 and Supplementary Video 1, the conventional CTM simulated substantial northward advective transport of pollutants from Shanxi and Shandong into the Beijing-Tianjin-Hebei (BTH) plain during January 12–15, a dynamic that was not corroborated by ground-based station observations. This quantitative tagging analysis confirms that the localized overestimation in Beijing primarily stemmed from the spatial accumulation and physical advection of upstream forecast errors (encompassing local emission inventory uncertainties, boundary-layer parameterization biases, and wind-field deviations). By explicitly resolving physical transport, ProNet successfully prevents these advected upstream errors from being misattributed to local chemical or emission sources in downwind urban receptors.

# Supplementary Section 10: Comparison of Coupled and all-AI Corrector Models

To justify the hybrid architecture of ProNet, we attempted an all-AI Corrector approach, where a neural network is used to directly correct the CTM outputs without being coupled with the physical transport module. We evaluated its performance and compared it with our proposed Coupled model. The results show that the all-AI Corrector approach—which utilizes CTM simulation data as a reference—achieves an overall forecast error (RMSE) of 17.8 μg m^-3^. This is significantly better than the Pure-AI model, demonstrating the clear benefit of incorporating CTM baseline information. Furthermore, in detailed case comparisons, the all-AI Corrector outperforms the Pure-AI model in terms of initial condition assimilation, simulation correlation coefficients, and extreme event forecasting. However, its performance still degrades compared to our proposed Coupled model (ProNet), which achieves a lower RMSE of 17.3 μg m^-3^ and exhibits superior capabilities across all aforementioned metrics. This comparative analysis explicitly justifies our design choice. The rationale for coupling a process-based transport module is that it can physically and accurately resolve the advection and diffusion of pollutants (i.e., transported errors). By analytically isolating these transported errors, the neural-network correctors can focus exclusively on estimating the newly generated uncertainties (e.g., emissions, deposition, and chemical reactions). An all-AI Corrector, lacking these explicit physical constraints, struggles to untangle complex, non-local transport dynamics from local source/sink uncertainties purely through data-driven learning (SI Fig. S17).

# Supplementary Section 11: Sensitivity Experiments on Training Data Scarcity

To evaluate the robustness and out-of-distribution generalization of forecasting models under limited training data availability, we conducted comprehensive sensitivity experiments using restricted training durations of 1, 3, 6, and 12 months. To rigorously challenge model generalization, the reduced training datasets (1-month, 3-month, and 6-month subsets) were strictly confined to low-pollution summer and autumn periods, leaving severe winter pollution regimes (specifically the heavily polluted month of January) entirely unobserved during model training. As illustrated in SI Fig. S20, while reducing training data from 12 months to 1 month expectedly elevates forecast errors across all architectures, the physics-coupled ProNet degrades at a significantly slower rate than purely data-driven models. Specifically, for ProNet, the overall forecast RMSE increases moderately from 14.9 to 22.1 μg m^-3^ (a 48% increase), whereas the Pure-AI model experiences a catastrophic performance degradation, with its RMSE surging from 16.3 to 29.2 μg m^-3^ (a 79% increase). During the unseen winter month of January, purely data-driven models completely fail to capture high-concentration pollution spikes, producing severe underestimations due to their inability to extrapolate beyond historical training distributions. In contrast, by grounding multi-step integrations within a process-based transport module, ProNet enforces physical advection constraints, thereby maintaining reliable spatial transport structures and capturing elevated pollution episodes even when trained on highly constrained, low-pollution datasets.

# Supplementary Section 12: Performance Benchmarking against Global AI Models

To benchmark ProNet against state-of-the-art global AI weather and atmospheric foundation models, we conducted a comprehensive evaluation comparing ProNet with Aurora across China for the full year of 2023. Because global models like Aurora do not produce continuous hourly outputs directly formatted for daily-mean comparisons, all model evaluations in this benchmark are evaluated on an hourly basis. The overall PM_2.5_ forecasting performance of Aurora yields an hourly RMSE of 32.6 μg m-3 across China, which is comparable to conventional CTM simulations (33.0 μg m-3). In comparison, our Pure-AI baseline achieves a lower hourly RMSE of 27.3 μg m-3, benefiting from a multi-time-step sequence input architecture and direct training on high-resolution localized surface data assimilation (incorporating 7,036 monitoring stations nationwide). By integrating physical transport constraints with data-driven error correctors, ProNet (Coupled model) further reduces the hourly forecast RMSE to 25.6 μg m-3, outperforming Aurora by 21% across all lead horizons (SI Fig. S5). Spatially, while global foundation models like Aurora exhibit localized accuracy advantages in specific northern boundary regions (such as Inner Mongolia) due to their incorporation of global satellite reanalysis products (CAMS), ProNet demonstrates overwhelming accuracy superiority across major population centers and key economic regions (including BTH, YRD, and PRD). This benchmarking highlights that while large global AI models excel at global synoptic patterns, coupling process-based chemical transport modules with localized neural correctors is essential for accurately capturing fine-scale atmospheric chemistry and complex surface pollution dynamics.

# Supplementary Section 13: Uncertainty Analysis of Interpretability Results

To evaluate whether ProNet’s interpretability outputs—temporal error apportionment and chemical mechanism diagnostics—are physically robust or dependent on model training artifacts, we conducted comprehensive sensitivity analyses using the 10 ensemble members derived from training epochs 291–300, as well as comparisons between target and predicted Uncertainty Errors.

Figure S14 presents the sensitivities of the temporal error apportionment under two perturbation sources: (1) substituting the target Uncertainty Error with 10 ensemble members’ predicted Uncertainty Errors, and (2) varying the neural corrector parameters across the 10 ensemble members. All results consistently demonstrate that initial condition errors exert diminishing influence on Beijing’s forecast errors beyond day 6, contributing less than 1% to the total error budget at extended lead times. The daily-mean error contributions across different ensemble members exhibit small standard deviations (less than 12%), confirming that the temporal attribution patterns are robust to training stochasticity. Comparing results obtained with the target Uncertainty Error versus the 10 predicted Uncertainty Errors reveals some sensitivity in the daily-mean error source partitioning, with differences in contribution ratios remaining below 31%. This indicates that while the precise numerical values carry inherent uncertainty, the qualitative temporal attribution patterns and their physical interpretations remain stable and reliable.

Figure S15 evaluates the stability of the SHAP-based chemical interpretability analysis across the 10 ensemble members. Specifically, the regression slope of nitrate against PM_2.5_ simulation errors fluctuates tightly within 5.1% (ranging from 0.057 to 0.060), and the slope for sulfate fluctuates within 19.7% (ranging from 0.160 to 0.195). These narrow fluctuation ranges confirm that the identified statistical associations between precursor components and CTM simulation errors are not artifacts of specific training instances.

# Supplementary Section 14: Systematic Out-of-Distribution Evaluation

To assess out-of-distribution generalization capabilities, we evaluated ProNet and the Pure-AI model, both trained exclusively on 2022 data and evaluated with observations from 2023. Following the lifting of pandemic lockdowns, PM_2.5_ concentrations in 2023 increased compared to 2022, providing a distinct data distribution to test the models. We stratified this evaluation across pollution levels, seasons, regions, and special events (SI Fig. S18).

**1. Stratification by Pollution Levels and Seasons:** In 2023, the occurrences of severe and beyond-severe pollution days increased by 63% and 45%, respectively, primarily due to higher spring and winter baseline concentrations. Under these higher pollution conditions, the Pure-AI model showed larger errors, with its accuracy on beyond-severe days being 6.8% lower than ProNet. Overall, ProNet performed better than the Pure-AI model across polluted categories, reducing the RMSE by 5.9 to 25.9 μg m^-3^ (SI Fig. S18a-f).

**2. Stratification by Region:** ProNet achieved a lower RMSE than the Pure-AI model in 97.3% of the evaluated cities. The RMSE reductions were observed across major regions, ranging from 0.7 μg m^-3^ in the PRD to 4.4 μg m^-3^ in the FWP (SI Fig. S18g-h).

**3. Special Events (Dust vs. Festivals):** For natural events like dust storms, ProNet assimilated upstream dust concentrations, resulting in an RMSE 4.3 μg m^-3^ lower than the Pure-AI model in BTHS during the dust period, compared to a 0.4 μg m^-3^ difference during non-dust spring periods. Conversely, during the Chinese New Year fireworks, both models had higher errors. The RMSE difference between ProNet and Pure-AI during the festival was similar to that on regular winter days (3.4 vs. 3.1 μg m^-3^), indicating that the single-year training dataset is insufficient for the models to capture sudden human-driven emission spikes without external domain knowledge (SI Fig. S18i-l).

# Supplementary Tables

**Table S1.** Input and output variables of deep neural networks experiment.

| **Variable** | **Description** | **Variable** | **Description** |
| --- | --- | --- | --- |
| **U** | Wind speed in the west to east direction | **Ter** | Terrain elevation |
| **V** | Wind speed in the south to north direction | **Land** | Land type |
| **windSpeed** | Wind speed | **Temp** | Temperature |
| **windDiv** | wind divergence | **RH** | Relative humidity |
| **PBLH** | Planetary boundary layer height | **ΔCO** | Changes of CO beside transportation in an hour |
| **PM_2.5_** | Particle matter with diameter lower than 2.5 μm | **ΔSO_2_** | Changes of SO_2_ beside transportation in an hour |
| **ΔPM_2.5_** | Changes of PM_2.5_ beside transportation in an hour | **ΔNO_2_** | Changes of NO_2_ beside transportation in an hour |
| **AER** | primary aerosol | **ΔNO** | Changes of NO beside transportation in an hour |
| **ΔAER** | Changes of primary aerosol beside transportation in an hour | **ΔC_2_H_6_** | Changes of C_2_H_6_ beside transportation in an hour |
| **ΔANH_4_** | Changes of ammonia beside transportation in an hour | **ΔISOP** | Changes of isoprene beside transportation in an hour |
| **ΔANO_3_** | Changes of nitrate beside transportation in an hour | **ΔHONO** | Changes of HONO beside transportation in an hour |
| **ΔASO_4_** | Changes of sulfate beside transportation in an hour | **ΔHNO_3_** | Changes of HNO_3_ beside transportation in an hour |
| **ΔSOA** | Changes of secondary organic aerosol beside transportation in an hour | **ΔHCHO** | Changes of HCHO beside transportation in an hour |
| **ΔOC** | Changes of organic carbon beside transportation in an hour | **ΔO_3_** | Changes of O_3_ beside transportation in an hour |
| **ΔNH_3_** | Changes of NH_3_ beside transportation in an hour | **O_3_** | Ozone |
| **PM_2.5__Err** | Error of forecasted PM_2.5_ by CTM | **ΔPM_2.5__Err** | Changes of PM_2.5__Err beside transportation in an hour |
| **c^m^** | Forecasted concentration of ensemble members in ProNet model | **c^p^** | Forecasted concentration of ensemble members in Pure-AI model |
| **y^w^** | Predicted ensemble weight by Ensemble Corrector | **y^b^** | Predicted ensemble bias by Ensemble Corrector |
| **y^E^** | Output of Emission-Deposition Corrector trying to predict the error caused by emission and deposition processes in CTM. | **y^R^** | Output of Chemical-Reaction Corrector trying to predict the error caused by chemical reactions in CTM. |

**Table S2.** Evaluated regions and cities.

| **Region** | **Province** | **Cities** |
| --- | --- | --- |
| **Beijing-Tianjin-Hebei and Surrounding Region** (BTHS, 38 cities) | Beijing | Beijing |
|  | Tianjin | Tianjin |
|  | Hebei | Shijiazhuang, Tangshan, Qinhuangdao, Handan, Xingtai, Baoding, Cangzhou, Langfang, Hengshui (9 cities in total) |
|  | Shandong | Jinan, Zibo, Zaozhuang, Dongying, Weifang, Jining, Tai’an, Rizhao, Linyi, Dezhou, Liaocheng, Binzhou, Heze (13 cities in total) |
|  | Henan | Zhengzhou, Kaifeng, Luoyang, Pingdingshan, Anyang, Hebi, Xinxiang, Jiaozuo, Puyang, Xuchang, Luohe, Sanmenxia, Shangqiu, Zhoukou (14 cities in total) |
| **Yangtze River Delta Region**  (YRD, 31 cities) | Shanghai | Shanghai |
|  | Jiangsu | Nanjing, Wuxi, Xuzhou, Changzhou, Suzhou, Nantong, Lianyungang, Huaian, Yancheng, Yangzhou, Zhenjiang, Taizhou, Suqian (13 cities in total) |
|  | Zhejiang | Hangzhou, Ningbo, Jiaxing, Huzhou, Shaoxing, Zhoushan (6 cities in total) |
|  | Anhui | Hefei, Wuhu, Bengbu, Huainan, Maanshan, Huaibei, Chuzhou, Fuyang, Suzhou, Lu’an, Bozhou (11 cities in total) |
| **Fenwei Plain**  (FWP, 13 cities) | Shanxi | Taiyuan, Yangquan, Changzhi, Jincheng, Jinzhong, Yuncheng, Linfen, Lüliang (8 cities in total) |
|  | Shaanxi | Xi’an, Tongchuan, Baoji, Xianyang, Weinan (5 cities in total) |
| **ChengYu Region** (CYR, 16 cities) | Chongqing | Chongqing |
|  | Sichuan | Chengdu, Zigong, Luzhou, Deyang, Mianyang, Suining, Neijiang, Leshan, Meishan, Yibin, Ya’an, Ziyang, Nanchong, Guang’an, Dazhou (15 cities in total) |
| **Yangtze River Midstream City Cluster**  (YRM,21 cities) | Hubei | Wuhan, Xianning, Xiaogan, Huanggang, Huangshi, Ezhou, Xiangyang, Yichang, Jingmen, Jingzhou (10 cities in total) |
|  | Jiangxi | Nanchang, Pingxiang, Xinyu, Yichun, Jiujiang (5 cities in total) |
|  | Hunan | Changsha, Zhuzhou, Xiangtan, Yueyang, Changde, Yiyang (6 cities in total) |
| **Pearl River Delta Region**  (PRD, 9 cities) | Guangdong | Guangzhou, Shenzhen, Zhuhai, Foshan, Jiangmen, Zhaoqing, Huizhou, Dongguan, Zhongshan (9 cities in total) |
| **Other Key Cities** (OKC, 40 cities) | Hebei, Shanxi, Shandong, Henan, Inner Mongolia, Liaoning, Jilin, Heilongjiang, Zhejiang, Anhui, Hubei, Fujian, Guangxi, Hainan, Guizhou, Yunnan, Tibet, Gansu, Qinghai, Ningxia, Xinjiang | Zhangjiakou, Chengde, Datong, Shuozhou, Xinzhou, Qingdao, Nanyang, Xinyang, Zhumadian, Hohhot, Baotou, Shenyang, Dalian, Chaoyang, Jinzhou, Huludao, Changchun, Harbin, Wenzhou, Jinhua, Quzhou, Taizhou, Lishui, Tongling, Anqing, Huangshan, Xuancheng, Chizhou, Suizhou, Fuzhou, Xiamen, Nanning, Haikou, Guiyang, Kunming, Lhasa, Lanzhou, Xining, Yinchuan, Ürümqi (40 cities in total) |

**Table S3.** Forecasting accuracy of PM_2.5_ in evaluated regions and cities.

| **Region** | | **Beijing** | **Shanghai** | **Guangzhou** | **BTHS** | **YRD** | **FWP** | **CYR** | **YRM** | **PRD** | **OKC** |
| --- | --- | --- | --- | --- | --- | --- | --- | --- | --- | --- | --- |
| 1-7 Day  Forecast | Human | 79.8% | 85.2% | 89.9% | 69.1% | 82.0% | 65.3% | 82.1% | 78.2% | 92.6% | 82.5% |
|  | Couple | 81.7% | 87.0% | 95.0% | 82.3% | 87.7% | 82.5% | 86.5% | 87.1% | 95.6% | 87.8% |
|  | Increase | 1.9% | 1.7% | 5.1% | 13.2% | 5.8% | 17.2% | 4.4% | 8.9% | 3.0% | 5.3% |
| 1 Day  Forecast | Human | 86.6% | 89.3% | 93.4% | 71.8% | 85.1% | 68.8% | 87.0% | 82.1% | 93.7% | 84.4% |
|  | Couple | 91.5% | 94.2% | 98.4% | 93.1% | 95.5% | 90.7% | 94.7% | 94.5% | 97.7% | 93.6% |
|  | Increase | 4.9% | 5.0% | 4.9% | 21.3% | 10.4% | 21.9% | 7.7% | 12.4% | 4.0% | 9.2% |
| 2 Day  Forecast | Human | 84.1% | 88.5% | 91.2% | 70.3% | 83.3% | 66.6% | 83.5% | 80.0% | 93.1% | 83.5% |
|  | Couple | 84.4% | 89.6% | 95.6% | 85.4% | 90.1% | 84.8% | 88.4% | 89.2% | 96.7% | 89.1% |
|  | Increase | 0.3% | 1.1% | 4.4% | 15.1% | 6.7% | 18.2% | 4.9% | 9.2% | 3.5% | 5.6% |
| 3 Day  Forecast | Human | 80.3% | 85.4% | 89.0% | 69.8% | 82.5% | 66.0% | 82.6% | 78.5% | 92.6% | 83.0% |
|  | Couple | 80.3% | 87.1% | 94.5% | 81.3% | 86.6% | 82.1% | 85.6% | 86.4% | 95.9% | 87.5% |
|  | Increase | 0.0% | 1.7% | 5.5% | 11.5% | 4.1% | 16.1% | 2.9% | 7.9% | 3.2% | 4.4% |
| 4 Day  Forecast | Human | 78.1% | 84.9% | 90.1% | 68.9% | 81.7% | 65.2% | 81.5% | 78.0% | 92.6% | 82.4% |
|  | Couple | 78.9% | 85.2% | 95.1% | 79.5% | 85.7% | 80.6% | 84.3% | 85.1% | 95.6% | 86.5% |
|  | Increase | 0.8% | 0.3% | 4.9% | 10.6% | 3.9% | 15.4% | 2.7% | 7.1% | 3.0% | 4.1% |
| 5 Day  Forecast | Human | 75.6% | 83.2% | 89.0% | 68.5% | 81.2% | 64.2% | 80.8% | 77.3% | 92.4% | 81.8% |
|  | Couple | 79.2% | 85.5% | 94.8% | 79.8% | 85.9% | 80.9% | 84.0% | 85.0% | 95.0% | 86.4% |
|  | Increase | 3.6% | 2.2% | 5.8% | 11.2% | 4.8% | 16.6% | 3.2% | 7.7% | 2.6% | 4.6% |
| 6 Day  Forecast | Human | 78.9% | 84.9% | 87.9% | 67.8% | 80.6% | 63.5% | 79.9% | 76.5% | 92.1% | 81.7% |
|  | Couple | 78.6% | 83.3% | 94.2% | 78.8% | 85.3% | 80.1% | 84.4% | 85.4% | 94.5% | 86.0% |
|  | Increase | -0.3% | -1.6% | 6.3% | 11.1% | 4.7% | 16.6% | 4.5% | 8.9% | 2.4% | 4.3% |
| 7 Day  Forecast | Human | 74.8% | 80.5% | 88.5% | 66.8% | 79.3% | 62.8% | 79.4% | 75.3% | 91.9% | 80.9% |
|  | Couple | 78.9% | 83.8% | 92.3% | 78.3% | 84.9% | 78.7% | 84.2% | 84.1% | 94.0% | 85.5% |
|  | Increase | 4.1% | 3.3% | 3.8% | 11.5% | 5.6% | 15.9% | 4.8% | 8.9% | 2.1% | 4.6% |

**Table S4** | IAQI and corresponding concentration limits

| **IAQI** | Daily average of PM_2.5_ |
| --- | --- |
| **0** | 0 |
| **50** | 30 |
| **100** | 75 |
| **150** | 115 |
| **200** | 150 |
| **300** | 250 |
| **400** | 350 |
| **500** | 500 |

# Supplementary Figures

Figure S1 Architecture of the Pure-AI model. In contrast to ProNet, this Pure-AI model removes all process-based modules and CTM inputs. a, Schematic of the Pure-AI forecasting framework. The outputs of Member Predictors are served as inputs for the Ensemble Predictor. Both predictors are trained using assimilated data as targets. b–c, Architectural schematics of the Member Predictor (b), Ensemble Predictor (c). Variable specifications are provided in Extended Data Table S1.

Figure S2 Domain configurations and distributions of monitoring sites. a, domain configurations for WRF and NAQPMS. The outer domain provides boundary conditions for the inner domain at a 45 km horizontal resolution of size 228×165. The inner domain covers the Chinese mainland at a 15 km horizontal resolution of size 464×300. b, map of the monitoring sites.

Figure S3 Performance advantages of the coupled model over purely process-based CTM. The coupled model quantifies and corrects Uncertainty Error – originating from emission estimates, chemical reactions, and deposition processes – at each CTM time step by deep learning modules. These modules are then coupled with the process-based transport module to correct the CTM forecasting error. a-c, Deep learning networks in the coupled model effectively capture the spatial and temporal patterns of the Uncertainty Error. a, Annual mean target Uncertainty Error in 2023. b, Annual mean predicted Uncertainty Error in 2023. c, Diurnal variation of Uncertainty Error across 2023 quarters. d-f, PM_2.5_ forecasts RMSE in 2023 from CTM (d), coupled model (e) and their differences (f), demonstrating improvements at 99.7% monitoring sites. g, Daily RMSE in 2023. h-i, Root mean square (RMS) of Uncertainty Error (h) and Total Error (i) for PM_2.5_ Forecasts, showing that the coupling framework could amplify the improvements by the data-driven networks, accumulating the correction of Uncertainty Error in each time step. j, PM_2.5_ forecast accuracy for China’s mainland and three key regions.

Figure S4 Dynamical consistency of the coupled modeling framework. a, Spatiotemporal evolution of PM_2.5_ concentrations in a representative forecast, showing abrupt, non-physical shifts in the purely AI-based model (first row) versus continuous pollutant transport governed by physical principles in the coupled model (second row) and the assimilated concentration (third row). b, Temporal evolution of regional mean PM_2.5_ concentrations in BTHS during the forecast period. c, Time series of the Index for Relative Mass Imbalance, evaluating relative mass conservation by calculating the ratio between the absolute difference in hourly concentration changes and the average CTM concentration within the evaluation region. d, Time series of the Index for Deviation of Temporal Variance, measuring temporal smoothness via normalized absolute differences in concentration second derivatives relative to the CTM. e, Time series of the Index of Transport Realism, evaluating adherence to atmospheric dynamics through the spatial Pearson correlation coefficient between target model hourly changes and purely physical transport-driven changes. The full dynamical evolution is shown in Supplementary Video 3.

Figure S5 Performance comparison of the Pure-AI model against Aurora over China. a and b are RMSE and R2 curves as functions of lead time for hourly PM_2.5_ forecasts across all monitored cities. c, Forecasting accuracy of PM_2.5_ for China as a whole (1) and three regions (2-4). d, Spatial distributions of RMSE difference between Pure-AI and Aurora. e and f are assimilated ICs for Pure-AI and Aurora, respectively. g, Seasonal RMSE by lead day. h, A case of forecast at Chifeng. i, A case of forecast at Beijing. Across these comparisons, the Pure-AI baseline generally shows lower RMSE and higher standardized forecast accuracy than Aurora over China in 2023.

Figure S6 Same as Fig. 5 but for O_3_

Figure S7 Spatial performance comparison of PM_2.5_ forecasts. a and b display RMSE maps for PM_2.5_ forecasts by human forecasters and ProNet, respectively. c shows the RMSE difference between ProNet and human forecasts. d, e and f present the analogous comparison for forecast accuracy, with d and e showing accuracy maps for human forecasters and ProNet, respectively, and f displaying their differences. ProNet demonstrates superior performance, reducing RMSE and enhancing forecast accuracy across most cities.

Figure S8 Same as Figure S7 but for O_3_

Figure S9 Validation of PM_2.5_ assimilation performance in 2022. a, Spatial distribution of annual mean PM_2.5_ concentrations derived from assimilated data in 2022. b-c, RMSE distribution before (b) and after (c) data assimilation. d-e, Time series comparison of observed and assimilated PM_2.5_ concentrations in Beijing (d) and Shanghai (e). Evaluation metrics, including RMSE, mean absolute error (MAE), correlation coefficient (R), and bias, are displayed in the upper right corner of each panel. Green and orange colors denote results before and after data assimilation, respectively.

Figure S10 Same as Figure S9 but for PM_2.5_ in 2023.

Figure S11 Time series and spatial distributions of the PM_2.5_ errors in air quality forecasting. Total forecast error is decomposed into Transported Error and Uncertainty Error through numerical transport analysis. a, c, and e present the spatial distributions of the Total Error, Transported Error, and Uncertainty Error, respectively. b, d, f and h display the corresponding temporal evolution across seasonal intervals: January-March, April-June, July-September, and October. g depicts the diurnal variation of Uncertainty Error. Notably, the Transported Error demonstrates spatiotemporal patterns nearly identical to Total Error, indicating that transport processes predominantly govern the short-term error dynamics in CTM forecasts of PM_2.5_ concentrations

Figure S12 Same as Figure S11 but for O_3_

**Figure S13 Cases of ensemble forecasts in the CYR, BTHS, YRD and PRD region, respectively.** Without ensemble correction, systematic biases from data-driven modules accumulate progressively, leading to an increase in ensemble spread in the first 3-4 days by orders of magnitude. Our ProNet approach constructs a 10-member ensemble using neural network parameters sampled from different training epochs; these are subsequently processed through the Ensemble Corrector to detect and mitigate bias accumulation, thereby improving long-term forecast accuracy.

Figure S14 Sensitivities of the temporal error apportionment. This figure shows the impacts of using target Uncertainty Error versus 10 ensemble members’ predicted Uncertainty Error on the temporal error apportionment. a-h present the error attribution to uncertainties in ICs and 1–7 day forecasts, respectively. All results consistently indicate that initial condition errors contribute less than 1% to Beijing’s forecast errors beyond day 6. The standard deviation of daily-mean error contributions across ensemble members is less than 12%, and the differences in contribution ratios between target and predicted Uncertainty Errors remain below 31%.

Figure S15 Sensitivities of the SHAP analysis by ensemble members. a and b present the SHAP dependence plots for sulfate and nitrate, respectively, evaluated across 10 ensemble members from training epochs 291–300. The regression slope of nitrate against PM_2.5_ simulation errors fluctuates within 5.1% (ranging from 0.057 to 0.060), and the slope for sulfate fluctuates within 19.7% (ranging from 0.160 to 0.195), confirming the statistical stability of the identified chemical associations.

Figure S16 Spatial distributions by CTM, Reanalysis and their difference in a PM_2.5_ forecast. a-d are the PM_2.5_ predicted by CTM. e-h are the CTM Error estimated by the difference between CTM and reanalysis data. i-l are the reanalysis data.

Figure S17 Same as Fig. 4 but replace Pure-AI model with all-AI Corrector. Pure-AI and the AI component in the all-AI Corrector have the same model structure. The Pure-AI directly predict future PM_2.5_ concentration, while the all-AI Corrector predict by correcting CTM forecasts with the AI component.

**Figure S18. Performance comparison of forecasting models across pollution levels, seasons, regions, and special events.** **a**, Change rate of city-days across different observed PM_2.5_ AQI levels from 2022 to 2023. **b-c**, Forecast accuracy (**b**) and RMSE (**c**) across different PM_2.5_ AQI levels. **d**, Averaged concentrations of observed and forecast PM_2.5_ by season. **e-f**, Forecast accuracy (**e**) and RMSE (**f**) by season. **g**, Spatial distribution of coupling-induced changes in RMSE across 338 Chinese cities. **h**, Forecast RMSE across different regions. **i**, Forecasts in China during the festival period. **j**, Forecasts in BTHS region during the dust event. **k-l**, Forecast RMSE during the festival (**k**) and dust (**l**) periods.

Figure S19 Sensitivity of training parameter on RMSE. a, RMSE of for 1-7day forecasts. b, Sensitivity of training parameters on the Coupled model. Base represents the training parameters adopted for the standard Couple model in this study. Three bars of the Base represent the results by three Ensemble Corrector with model parameters from epoch 150, 200 and 250. LR_L and LR_H represents the results by model with lower and higher learning rates, respectively. En_L and En_H represent the results by models with ensemble from lower and higher epochs of Correctors in ETM. En_S represent the results by models with smaller ensemble size.

Figure S20 Impact of training datasets on model performance. a and b present the RMSE and Bias of model with different training datasets. As training data decreases from 12 months down to 1 month, the overall forecast skill decrease. The Coupled model (ProNet) degrades at a slower rate than the Pure-AI model, indicating stronger generalization.

Figure S21 Decreases of RMSE by components in ProNet. Under the ProNet architecture, the process-driven transport module could improve forecasts by correcting the errors from ICs, which is evaluated by the forecasts of CTM + ETM without AI Correctors. The Correctors in ETM further correct the uncertainties during forecasts, which is evaluated by Couple without the Ensemble Corrector. The Ensemble Corrector further suppresses accumulation of error stemming from the Correctors in ETM. The Pure-AI directly predict future PM_2.5_ concentration, while the all-AI Corrector predict by correcting CTM forecasts.

**References**

1.	Chen L, Gao Y, Zhang M *et al.* MICS-Asia III: multi-model comparison and evaluation of aerosol over East Asia. *Atmos Chem Phys*. 2019; **19**: 11911–11937. doi: 10.5194/acp-19-11911-2019

2.	Li M, Zhang Q, Kurokawa J-i *et al.* MIX: a mosaic Asian anthropogenic emission inventory under the international collaboration framework of the MICS-Asia and HTAP. *Atmos Chem Phys*. 2017; **17**: 935–963. doi: 10.5194/acp-17-935-2017

3.	Crippa M, Solazzo E, Huang G *et al.* High resolution temporal profiles in the Emissions Database for Global Atmospheric Research. *Sci Data*. 2020; **7**: 121. doi: 10.1038/s41597-020-0462-2

4.	Nenes A, Pandis SN, Pilinis C. ISORROPIA: A New Thermodynamic Equilibrium Model for Multiphase Multicomponent Inorganic Aerosols. *Aquat Geochem*. 1998; **4**: 123–152. doi: 10.1023/a:1009604003981

5.	Seinfeld JH, Pandis SN. *Atmospheric Chemistry and Physics: From Air Pollution to Climate Change*. Hoboken, NJ: Wiley, 2016.

6.	Keller CA, Evans MJ. Application of random forest regression to the calculation of gas-phase chemistry within the GEOS-Chem chemistry model v10. *Geosci Model Dev*. 2019; **12**: 1209–1225. doi: 10.5194/gmd-12-1209-2019

7.	Walcek CJ, Aleksic NM. A simple but accurate mass conservative, peak-preserving, mixing ratio bounded advection algorithm with FORTRAN code. *Atmos Environ*. 1998; **32**: 3863–3880. doi: 10.1016/s1352-2310(98)00099-5

8.	Wang Z, Maeda T, Hayashi M *et al.* A Nested Air Quality Prediction Modeling System for Urban and Regional Scales: Application for High-Ozone Episode in Taiwan. *Water Air Soil Pollut*. 2001; **130**: 391–396. doi: 10.1023/a:1013833217916

9.	Byun DW, Dennis R. Design artifacts in eulerian air quality models: Evaluation of the effects of layer thickness and vertical profile correction on surface ozone concentrations. *Atmos Environ*. 1995; **29**: 105–126. doi: 10.1016/1352-2310(94)00225-a

10.	Zaveri RA, Peters LK. A new lumped structure photochemical mechanism for large-scale applications. *J Geophys Res-Atmos*. 1999; **104**: 30387–30415. doi: 10.1029/1999jd900876

11.	Odum JR, Jungkamp TPW, Griffin RJ *et al.* The Atmospheric Aerosol-Forming Potential of Whole Gasoline Vapor. *Science*. 1997; **276**: 96–99. doi: 10.1126/science.276.5309.96

12.	Pandis SN, Harley RA, Cass GR *et al.* Secondary organic aerosol formation and transport. *Atmos Environ a-Gen*. 1992; **26**: 2269–2282. doi: 10.1016/0960-1686(92)90358-r

13.	Li J, Wang Z, Zhuang G *et al.* Mixing of Asian mineral dust with anthropogenic pollutants over East Asia: a model case study of a super-duststorm in March 2010. *Atmos Chem Phys*. 2012; **12**: 7591–7607. doi: 10.5194/acp-12-7591-2012

14.	Wesely ML. Parameterization of surface resistances to gaseous dry deposition in regional scale numerical models. *Atmos Environ*. 1989; **23**: 1293–1304. doi: 10.1016/0004-6981(89)90153-4

15.	Stockwell WR, Middleton P, Chang JS *et al.* The second generation regional acid deposition model chemical mechanism for regional air quality modeling. *J Geophys Res-Atmos*. 1990; **95**: 16343–16367.

16.	Cao GL, Zhang XY, Wang D *et al.* Inventory of atmospheric pollutants discharged from biomass burning in China continent (in Chinese). *China Environ Sci*. 2005; **25**: 389–393.

17.	Guenther A, Karl T, Harley P *et al.* Estimates of global terrestrial isoprene emissions using MEGAN (Model of Emissions of Gases and Aerosols from Nature). *Atmos Chem Phys*. 2006; **6**: 3181–3210.

18.	Athanasopoulou E, Tombrou M, Pandis SN *et al.* The role of sea-salt emissions and heterogeneous chemistry in the air quality of polluted coastal areas. *Atmos Chem Phys*. 2008; **8**: 5755–5769. doi: 10.5194/acp-8-5755-2008

19.	Luo G, Wang Z. A global environmental atmospheric transport model (GEATM): Model Description and validation (in Chinese). *Chin J Atmos Sci*. 2006; **30**: 504–518.

20.	Powers JG, Klemp JB, Skamarock WC *et al.* The Weather Research and Forecasting Model: Overview, System Efforts, and Future Directions. *Bull Am Meteorol Soc*. 2017; **98**: 1717–1737.

21.	Allen-Zhu Z, Li YZ, Liang YY. Learning and Generalization in Overparameterized Neural Networks, Going Beyond Two Layers. In: Advances in Neural Information Processing Systems 32. Curran Associates, Inc., 2019, 6155–6166.

22.	Smagorinsky J. General circulation experiments with the primitive equations: I. The basic experiment. *Mon Weather Rev*. 1963; **91**: 99–164.

23.	Evensen G. The ensemble Kalman filter: Theoretical formulation and practical implementation. *Ocean Dyn*. 2003; **53**: 343–367.

24.	Houtekamer PL, Mitchell HL. A Sequential Ensemble Kalman Filter for Atmospheric Data Assimilation. *Mon Weather Rev*. 2001; **129**: 123–137. doi: 10.1175/1520-0493(2001)129<0123:Asekff>2.0.Co;2

25.	Gaspari G, Cohn SE. Construction of correlation functions in two and three dimensions. *Q J R Meteorol Soc*. 1999; **125**: 723–757. doi: 10.1002/qj.49712555417

26.	Lundberg SM, Lee S-I. A Unified Approach to Interpreting Model Predictions. In: Guyon I, Luxburg UV, Bengio S, et al. Advances in Neural Information Processing Systems 30. Curran Associates, Inc., 2017, 4765–4774.

27.	Baydin AG, Pearlmutter BA, Radul AA *et al.* Automatic differentiation in machine learning: a survey. *J Mach Learn Res*. 2018; **18**: 1–43.
